# Engineering protein theranostics using bio-orthogonal asparaginyl peptide ligases

**DOI:** 10.7150/thno.53615

**Published:** 2021-04-03

**Authors:** Zhen Wang, Dingpeng Zhang, Xinya Hemu, Side Hu, Janet To, Xiaohong Zhang, Julien Lescar, James P. Tam, Chuan-Fa Liu

**Affiliations:** School of Biological Science, Nanyang Technological University, 60 Nanyang Drive, Singapore 637551.

**Keywords:** protein theranostics, protein labelling, peptide asparaginyl ligases, bio-orthogonal ligation

## Abstract

**Background:** Protein theranostics integrate both diagnostic and treatment functions on a single disease-targeting protein. However, the preparation of these multimodal agents remains a major challenge. Ideally, conventional recombinant proteins should be used as starting materials for modification with the desired detection and therapeutic functionalities, but simple chemical strategies that allow the introduction of two different modifications into a protein in a site-specific manner are not currently available. We recently discovered two highly efficient peptide ligases, namely butelase-1 and VyPAL2. Although both ligate at asparaginyl peptide bonds, these two enzymes are bio-orthogonal with distinguishable substrate specificities, which can be exploited to introduce distinct modifications onto a protein.

**Methods:** We quantified substrate specificity differences between butelase-1 and VyPAL2, which provide orthogonality for a tandem ligation method for protein dual modifications. Recombinant proteins or synthetic peptides engineered with the preferred recognition motifs of butelase-1 and VyPAL2 at their respective C- and N-terminal ends could be modified consecutively by the action of the two ligases.

**Results:** Using this method, we modified an EGFR-targeting affibody with a fluorescein tag and a mitochondrion-lytic peptide at its respective N- and C-terminal ends. The dual-labeled protein was found to be a selective bioimaging and cytotoxic agent for EGFR-positive A431 cancer cells. In addition, the method was used to prepare a cyclic form of the affibody conjugated with doxorubicin. Both modified affibodies showed increased cytotoxicity to A431 cells by 10- and 100-fold compared to unconjugated doxorubicin and the free peptide, respectively.

**Conclusion:** Bio-orthogonal tandem ligation using two asparaginyl peptide ligases with differential substrate specificities is a straightforward approach for the preparation of multifunctional protein biologics as potential theranostics.

## Introduction

By combining therapy with the specific diagnostic information of a disease target, theranostics promises to optimize the efficacy and safety of precision medicine 1-3. This has led to a tremendous interest in the development of theranostic agents for the treatment of cancer 4, 5. In addition to the use of nanomedicine platforms for the development of theranostics 6-9, a molecular-based approach involves the attachment of imaging agents and cytotoxic drugs to cancer-targeting proteins and antibodies 10, 11. In particular, small protein ligands, such as antibody fragments and mimetics, offer the advantages of low production cost, good tissue penetration, and easy maneuverability for designing end products with defined chemical composition. However, a major challenge in developing protein-based theranostic agents lies in the conjugation of the protein ligand with the imaging and treatment moieties 10. Clearly, the conjugation strategy should be able to introduce at least two modifications onto a protein substrate. Although numerous chemical techniques have been developed for protein labeling 12, 13, a simple strategy that allows for two consecutive site-specific modifications to be performed on a straight recombinant protein has yet to be developed.

Owing to their high specificity and mild operating conditions, biosynthetic methods that modify proteins through special recognition tags are attractive alternatives to chemical methods 14. The main advantage of these tag-mediated protein labeling methods is that the tags are themselves a peptide segment or protein domain and thus can be genetically fused to the protein of interest (POI). Of these methods, those that are based on peptide ligases are of utmost interest. Peptide ligases catalyze the formation of new peptide bonds between ligation partners, which makes them particularly useful bioconjugation tools for protein-based theranostics. Notable examples of peptide ligases include subtiligase 15-19, sortase A 20-24, and butelase-1 25-29, which are all tag-recognizing enzymes and can label proteins specifically at the terminal ends. Subtiligase is an artificially engineered ligase that uses an ester or thioester tag for protein labeling 15-19. Sortase A requires a 5-residue tag, LPETG, and catalyzes transpeptidation at the Thr residue 20-24. However, the use of the 5-residue tag notwithstanding, the enzymatic activity of sortase A is very low. Butelase-1 is a peptidyl asparaginyl ligase or PAL. So far, the most powerful peptide ligases have been found in the PAL family, and the most efficient PAL is butelase-1. Structurally, butelase-1 is a member of the commonly known asparaginyl endopeptidase (AEP) or legumain family 30, 31. Depending on the pH or substrate, certain AEPs are also found to display PAL activities 32-42. Butelase-1 is unique in that it functions almost as a pure PAL with no protease activity at weakly acidic to weakly basic pH. It has been shown to catalyze protein and peptide ligation with a high specificity and efficiency 25-29. Like all PALs, butelase-1 recognizes a short tripeptide tag, such as NHV, and cleaves the peptide bond at Asn to rejoin it with the amino terminal residue of another peptide. Thus, only an Asn residue is left in the ligation product, making butelase-mediated ligation (BML) nearly traceless. This is in significant contrast to most of the above-mentioned biosynthetic methods, which leave a large “scar” in the modified protein 14. Recently, VyPAL2, another plant legumain from the *Viola Yedoensis* family, was identified as a highly active PAL 42. Its catalytic efficiency was 274,325 M^-1^·s^-1^ in the cyclization of a model peptide, making it one of the fastest PAL reported to date 42. In addition, the proenzyme of VyPAL2 can be readily expressed in insect cells and can be self-processed at acidic pH to yield the active enzyme 42. These features make VyPAL2 a very attractive ligase for protein labeling 43. As asparaginyl transpeptidases, both butelase-1 and VyPAL2 use their active-site cysteinyl thiol group to cleave an Asn-Xaa peptide bond in their acyl-donor substrate, forming an acyl-enzyme thioester intermediate. Instead of being attacked by a water molecule for hydrolysis, as in the case of an asparaginyl endopeptidase, the acyl-enzyme thioester intermediate undergoes aminolysis by a peptidic nucleophile, which results in the formation of a new asparaginyl peptide bond between the acyl-donor substrate and the nucleophile substrate. Intriguingly, there seem to be noticeable differences in substrate specificity between VyPAL2 and butelase-1. VyPAL2 has a relatively low activity towards the tripeptide NHV, which, on the other hand, is one of the preferred recognition motifs of butelase-1 25, 42. In addition, a nucleophile peptide with a Phe at the P2″ position is a weak substrate for butelase-1 25, but it is favored by VyPAL2 42. We reasoned that these differential substrate specificities might provide sufficient orthogonality for a tandem ligation strategy for protein dual labeling.

**Figure 1:**
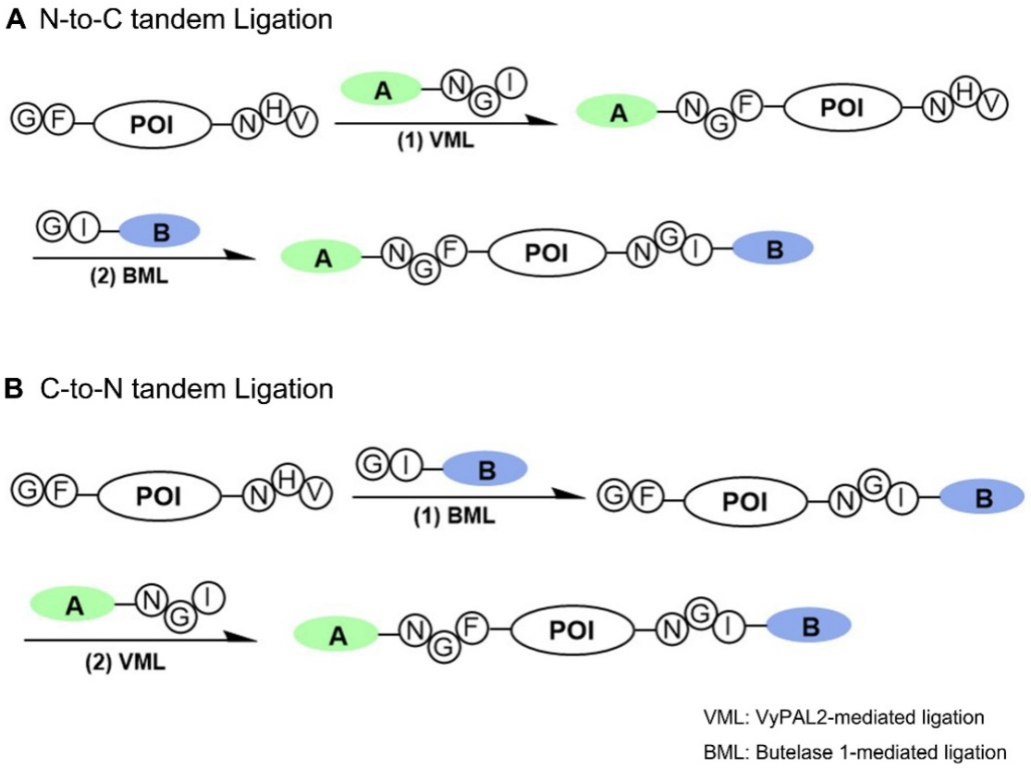
Bi-directional dual protein labeling by bio-orthogonal tandem ligation in N-to-C (A) or C-to-N (B) direction using butelase-1 and VyPAL2.

Several protein dual modification methods involving the use of peptide ligases have been reported 44-49. For example, consecutive protein modifications were achieved chemoenzymatically by combining chemoselective conjugation and sortase A- or butelase-1-mediated ligation 44-46. Two sortases of different substrate specificities were used to label a protein at both the N- and C-termini 49. Butelase-1 was also used together with sortase A for protein dual labeling in a three-step scheme 46. The two enzymes were also used for one-pot dual labeling of an antibody at the respective C-terminal ends of light and heavy chains 47. These last two schemes are bio-orthogonal, taking advantage of the distinct substrate specificity of two completely different ligases. However, as discussed above, owing to its extremely slow kinetics and relatively long recognition tag, the use of sortase A has its inherent limitations. Recently, an interesting method was reported that allowed two consecutive ligation reactions on the same protein substrate from the C- to N-terminus direction 48. However, it should be noted that this scheme is semi-orthogonal because it requires the protection of the protein's N-terminal amine by a TEV recognition sequence during the first ligation step to avoid the cyclization or self-ligation of the protein substrate 48. Here, we reported a bio-orthogonal scheme using two asparaginyl peptide ligases - butelase-1 and VyPAL2 - which allows for tandem ligation on the same protein in either the N-to-C or C-to-N direction, leading to its dual labeling at the C- and N-terminal ends (Figure 1). No protection on the protein substrate is required when performing the first ligation step, although butelase-1 and VyPAL2 are both asparagine-specific. Thus, a distinct advantage of bio-orthogonal ligation is the use of mild enzymatic reactions under aqueous conditions, which are compatible with biologics, such as proteins, antibodies and live cells.

**Figure 2:**
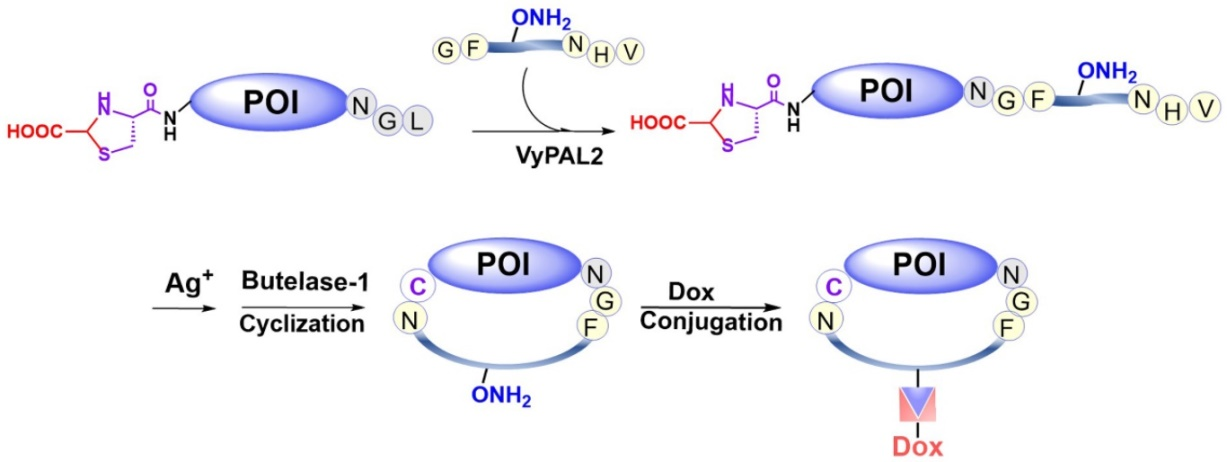
Preparation of a cyclic affibody-drug conjugate by PAL-mediated tandem ligation-cyclization and drug conjugation.

In addition to N- and C-terminal directed protein dual labeling, our bio-orthogonal tandem ligation strategy can also be used to prepare a cycloprotein-drug conjugate or cPDC (Figure 2). This involves the use of a synthetic intervening peptide designed to join the two termini of a protein. The peptide is trifunctional, containing an N-terminal GF-dipeptide nucleophile substrate for VyPAL, a C-terminal NHV tripeptide motif as the acyl donor substrate for butelase-1 and an internal aminooxy functionality for oxime conjugation, which would allow consecutive PAL-mediated ligation, cyclization, and doxorubicin attachment (Figure 2). Given the expected thermal and metabolic stability of cycloproteins, cycloprotein conjugates are interesting candidates for theranostics.

Using an EGFR-binding affibody 50, 51 as the model protein, we demonstrated the feasibility of our tandem ligation strategy for the preparation of dually labeled proteins and cycloprotein-drug conjugates. Because butelase-1 and VyPAL2 are the two most powerful ligases, such a bio-orthogonal tandem ligation strategy would offer an ideal solution to the challenging problem of manufacturing protein-based theranostics and other biologics with unusual architectures and functionalities.

## Methods

All amino acids, coupling reagents, solvents, and resins were purchased from Sigma and Chemimpex. All solvents and reagents were used as received without further purification. VyPAL2 and butelase-1 were prepared in-house, as previously reported.

### HPLC

Analytical RP-HPLC was run on a SHIMADZU (Prominence LC-20AT) instrument using an analytical column (Grace Vydac “Protein C4”) (250 × 4.6 mm, 5 µm particle size) at a flow rate of 1.0 mL/min. Analytical HPLC elution was monitored by UV absorption at 214 nm and 254 nm. Semi-preparative RP-HPLC was run on a SHIMADZU (Prominence LC-20AT) instrument using a semi-preparative column (Grace Vydac “Protein C4”) (250 × 10 mm, 10 µm particle size) at a flow rate of 2.5 mL/min. Both analytical and semi-preparative HPLC were run at room temperature using a gradient of solvent B in solvent A. Solvent B was 90% acetonitrile in water (0.040% TFA) and solvent A was water (0.045% TFA). Both solvents were filtered through 0.22-µm filter paper and sonicated for 30 min before use.

### Protein expression and purification

Genes encoding the desired protein sequences were cloned into the pETDuet vector and the plasmids were then transformed into *E. coli* BL21 (DE3) competent cells using the standard 90 s heat shock protocol. The bacterial colonies were then transferred to liquid LB medium in a culture flask. The flask was shaken in an incubator at 37 °C for 8-12 h until the OD reached 0.6-0.8, followed by induction with 1 mM IPTG at 37 °C for 4-8 h for protein expression. Cells were harvested and lysed by sonication in lysis buffer containing 50 mM sodium phosphate and 500 mM NaCl (pH 8.0). After centrifugation, the supernatant was loaded onto a column of Ni-NTA beads and incubated at 4 °C for 1 h. The beads were washed three times with lysis buffer, and the protein was subsequently eluted with lysis buffer containing 250 mM imidazole. The purified protein was dialyzed in phosphate buffer (pH 6.5) overnight and stored in a freezer at -20°C.

### Mass spectrometry

The ESI mass spectrum data of small peptides and proteins were obtained from a Thermo Finnigan LCQ DECA XP MAX (ESI ion source, positive mode). MagTran 1.03 and ESIProt 1.0 software were used for data deconvolution.

### Tissue culture and cell imaging

Cells were maintained in 10% FBS in DMEM (high glucose) at 37 °C in an incubator under 5% CO_2_. For passaging, cells were first washed three times with trypsin-EDTA (0.25%) to detach the cells from the tissue culture plates. Then, a 3-fold volume of complete DMEM medium was added to neutralize trypsin activity. Cells were grown until 40-60% confluency. Peptides or proteins in complete medium were applied to the cells and incubated for 30 min at 37 °C. Washing was performed three times with PBS, and the cells were subsequently subjected to microscopy analysis.

### Cell viability assay

MTT assays were carried out following the recommended protocols from Sigma-Aldrich (Cat. No. 11465001001). First, the cells were seeded in a 96-well tissue culture plate with 100 µL medium to grow until the confluency reached 40-60% of the plate surface. Peptides and proteins were added and incubated for 84 h, followed by the addition of 10 µL of MTT I to each well before incubating further for approximately 4 h. Next, MTT II was added and incubated at 37 °C overnight to solubilize the purple crystals. Spectrophotometric absorbance measurements of the samples were carried out using a microplate reader (Biotek, citation 5) at a wavelength of 575 nm; the reference wavelength was 670 nm.

### Cell staining and imaging

MCF-7 and A431 cells cultured in 24-well plates were washed three times with PBS. Formaldehyde (4%, w/v in PBS) was then added to each well for 15 min to fix the cells. Then, the cells were washed with PBS three times to remove residual formaldehyde. To permeabilize the cells, Triton X-100 (0.1%, w/v in PBS) was added to the wells for 5 min. Then, PBS was used to wash the cells another three times before staining. To stain the cells, doxorubicin, protein **26**, and DAPI were diluted in PBS to a concentration of 10 μM, 2 μM, and 700 nM, respectively. Then, the solution was added to each well for 30 min. Next, the cells were washed with PBS three times and subjected to imaging analysis using inverted fluorescence microscopy (#IX71; Olympus Life Science). To acquire the DAPI fluorescent image, the “Blue” channel (filter cube: 350 nm) was used. Likewise, the “Red” channel (filter cube: 550 nm) was used to obtain the doxorubicin fluorescence, while the “Green” channel (Filter Cube: 450 nm) was used for fluorescein.

### Solid-phase peptide synthesis

All peptides used in this study were synthesized as C-terminal amides using Rink amide MBHA resin by standard Fmoc chemistry. Before use, the resin was pre-swelled in DMF for 20 min. Before the first coupling, an Fmoc deprotection procedure was performed using 20% piperidine in dimethylformamide (DMF) for 30 min. The resin was then washed successively with DMF, DCM, and DMF. For the coupling reactions, 3 equiv. of Fmoc-AA-OH and 3 equiv. of PyBOP were dissolved in DMF/DCM (1:1). This mixture was added to the resin, followed by the addition of 6 equiv. of DIEA. Coupling reactions were carried out for 60-90 min. Coupling efficiency was evaluated using the ninhydrin test. For peptides **14** and **19**, Fmoc-Lys(Biotin)-OH was used. For peptide **23**, after peptide assembly on the solid phase, the Boc group on the lysine side chain amine was removed with 1 M HCl in DCM for 30-40 min, then 5(6)-carboxyfluorescein was coupled to the side-chain free amine using PyBOP as the coupling reagent. The peptides were cleaved from the resin with a cocktail containing 95% TFA, 2.5% water, and 2.5% TIS for 2 h. After precipitation with cold diethyl ether, the crude peptides were purified using HPLC. The desired peptides were obtained in powder form after lyophilization. All peptides were characterized by electrospray ionization mass spectrometry.

List of peptides prepared in the study (letters in lower case denote D-amino acids):

- Peptide **1**: Ac-**KKLAVINHV**; 1061.01 (observed), 1062.27 (calculated).
- Peptide **2**: **GIGGIKA**; 613.68 (observed), 613.74 (calculated).
- Peptide **4**: **YKANGL**; 664.26 (observed), 664.67 (calculated).
- Peptide **5**: **GFGGIKA**; 648.38 (observed), 648.52 (calculated).
- Peptide **7**: Ac**-KKLAVINGF**; 1031.34 (observed), 1031.56 (calculated).
- Peptide **9**: Fluorescein-**GRANGI**; 944.52 (observed), 944.97 (calculated).
- Peptide **11**: **GIGGFKGG**-**klaklakklaklak**; 2197.07 (observed), 2197.72 (calculated).
- Peptide **14**: **HVGGRIK**(Biotin)**GA**; 1119.89 (observed), 1118.61 (calculated).
- Peptide **19**:** GVGGRIK**(Biotin)**GA**; 1039.61 (observed), 1038.58 (calculated).
- Peptide **23**: **GIGGIRK**(Fluorescein); 1057.65 (observed), 1057.35 (calculated).
- Peptide **25**: **GFLGVK**(COCH_2_ONH_2_)**ANHV**; 1113.90 (observed), 1113.29 (calculated).

The numbering and illustrative structures of peptides and proteins prepared in this study are shown in Figure S1.

## Results and Discussion

### Differential substrate specificity of butelase-1 and VyPAL2 analyzed by kinetic studies

PAL enzymes have been used extensively for protein single-site labeling and macrocyclization. However, the use of two PALs with different substrate specificities for bio-orthogonal and dual ligation remains unexplored. Previous studies have revealed noticeable differences in substrate specificity between butelase-1 and VyPAL2 25, 42. To evaluate these differences quantitatively, we first studied the kinetics of VyPAL2 and butelase-1 toward peptide **1**, which has a C-terminal NHV tripeptide motif (Table 1). The nucleophile substrate, used at a constant concentration for kinetic studies, was peptide **2**, which contains an N-terminal GI dipeptide motif. Reverse-phase analytical HPLC was used to monitor and quantify the ligation reaction. Our results showed that, in this ligation reaction, the catalytic activity of butelase-1 towards acyl peptide substrate **1** was approximately 18.5 times that of VyPAL2 (Table 1 and Figure S2). Similarly, the kinetics of the two ligases towards a GF-starting nucleophile substrate, peptide **5**, were also examined. In this case, the acyl substrate, which was kept at a constant concentration for kinetic studies, was peptide **4**, which contains NGL at the C-terminus, a favorable motif for both VyPAL2 and butelase-1. VyPAL2 was found to be 4.6-fold more efficient than butelase-1 towards GF-peptide substrate **5** (Table 1 and Figure S2). We also conducted kinetic studies on another acyl donor substrate, peptide **7**, and found that butelase-1 exhibited catalytic activity towards this NGF peptide which was 6.5-fold lower than that of NHV peptide **1**. The difference in catalytic efficiency between VyPAL2 and butelase-1 towards NGF substrate **7** was about 2.7-fold.

An analysis of the butelase-1 and VyPAL2 structures helps to explain their differential substrate specificities, which are likely due to differences in the S1′ and S2′ substrate binding pockets of the two enzymes 41, 42. For butelase-1, a glycine residue (Gly167) and a valine residue (Val170) occupy the central positions of its S1′ and S2′ pockets, respectively. However, for VyPAL2, the same spots are occupied by alanine (Ala174) and lysine (Lys177), respectively. The small Gly residue in the S1′ pocket of butelase-1 makes it possible for the enzyme to tolerate a variety of amino acid residues at the P1′ position of its substrates. The Val residue in its S2′ pocket makes it prefer these P2′-amino acids of its substrates with a bulky aliphatic side chain for van der Waals interactions. On the other hand, VyPAL2 has an Ala in its S1′ pocket, which is larger than Gly. This may hinder the binding of a substrate with a larger amino acid (e.g., His) at the P1′ position. The long aliphatic side chain and the positive charge of the lysine residue in the S2′ pocket of VyPAL2 may explain that the enzyme can accept a P2′ (or P2″) residue like Ile or Leu, which has a large aliphatic side chain for attraction by Van der Waals forces, or Phe, which can interact with S1′-Lys through cation-π interactions. In summary, the kinetic studies confirm the differential activities of butelase-1 and VyPAL2 toward certain substrate sequences, providing strong support for a two-PAL, bio-orthogonal tandem ligation scheme for protein dual labeling.

### Applying the two PAL-based bio-orthogonal tandem ligation method for affibody dual labeling

**Figure 3:**
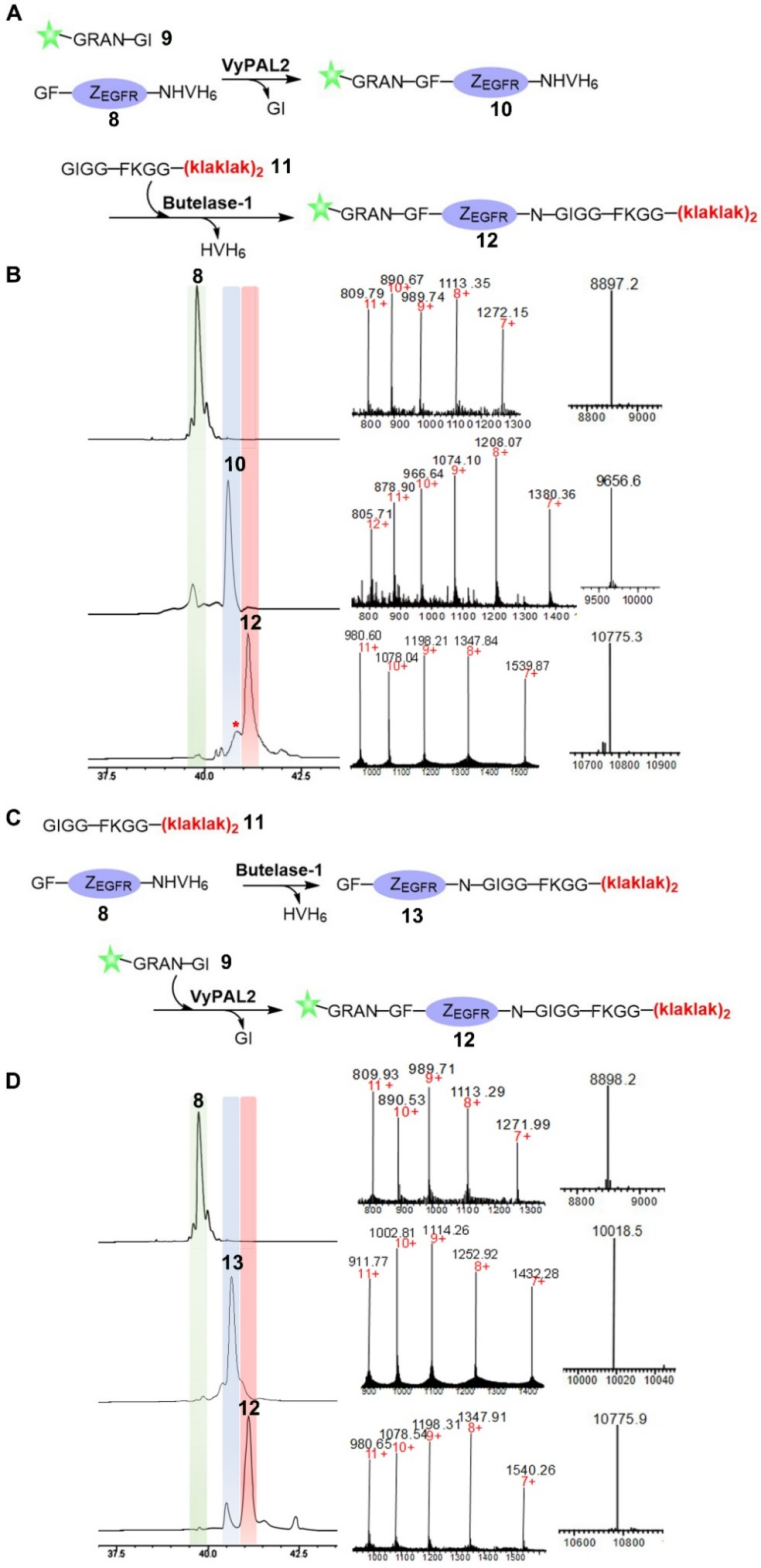
Bio-orthogonal protein dual labeling using VyPAL2 and butelase-1. (A) N-to-C tandem ligation scheme. Fluorescein-peptide **9** was first ligated to the N terminus of Z_EGFR_ **8** via VML to give **10** which was then ligated with peptide **11** at C terminus via BML to give **12**; (B) HPLC and LC-MS analysis of N-to-C ligation. The ligation products **8**, **10**, **12** were purified by reverse-phase HPLC and analyzed via ESI-MS; (C) C-to-N tandem ligation scheme. Mitochondrion-lytic peptide **11** is conjugated at C terminus of Z_EGFR_ **8** to give **13** via BML and then the fluorescein-peptide **9** is ligated to the N terminus of **13** to produce **12**; (D) HPLC and LC-MS analysis of N-to-C ligation. The ligation products **8**, **13**, **12** were purified by HPLC and analyzed by ESI-MS (**8:** calcd 8896.8, obsvd 8897.2; **10:** calcd 9652.6, obsvd 9656.6; **12**: calcd 10774.3, obsvd 10775.3 or 10775.9; **13**: calcd 10017.8, obsvd 10018.5). Details of by-product characterization are provided in Supporting Information.

Next, we proceeded to use butelase-1 and VyPAL2 to dually label the affibody through tandem enzymatic ligation (Figure 3). Considering the specificity of the two enzymes, an N-terminal GF dipeptide tag and a C-terminal NHV tripeptide tag were introduced onto Z_EGFR_ to obtain **8**. A new fluorescein-peptide **9** with a C-terminal NGI motif was prepared. We also synthesized peptide **11**, GIGGFKGG-klaklakklaklak, of which the all-D amino-acid sequence is the mitochondrion-lytic **KLA** peptide 52. Phe-Lys is a cathepsin B-sensitive linker 53 and can be cleaved in the lysosomes to release the **KLA** peptide. Peptides **9** and **11** were used to label the respective N- and C-termini of the Z_EGFR_ **8**. Sequential bio-orthogonal ligations were conducted in both the N-to-C (Figure 3A) and C-to-N (Figure 3C) directions. For N-to-C tandem ligation, VyPAL2 was used at the first ligation step, while butelase-1 was used at the second step (Figure 3A). C-to-N sequential ligations were performed using the two enzymes in the reverse order (Figure 3C). Whichever the ligation direction, the same final product **12** was obtained, as characterized by ESI-MS (obs: 10776, calc: 10773). The reactions at each step of the two schemes were remarkably clean with good conversion yields. In N-to-C ligation, 50 μM of Z_EGFR_ **8** and 250 μM of peptide **9** were first reacted in the presence of 150 nM of VyPAL2 at 37 °C for 30 min. The reaction gave *ca.* 80% of product **9** based on HPLC analysis. After HPLC purification and refolding, the second step was performed by incubating 50 μM of **10** and 250 μM of peptide **11** with 100 nM of butelase-1 for 20 min at 37 °C. The second step resulted in a conversion yield of around 70% (Figure 3B). A small amount of a by-product (~10%) was found in the second step (Figure 3B and S3). This was because, although the newly formed NGF motif in **10** was not a favored substrate of butelase-1, its 6.5-fold lower reactivity than the NHV motif (Table 1) meant that it could still be affected in BML, which resulted in the cleavage of the N-G peptide bond for transpeptidation with **11**. In C-to-N ligation, BML was first performed by mixing 50 μM of Z_EGFR_ **8** and 250 μM of peptide **11** with 100 nM of butelase-1 at 37 °C for 30 min to obtain **13** in approximately 85% based on HPLC analysis. Then, VML was performed by incubating 50 μM of purified **13** and 250 μM of peptide **9** with 100 nM of VyPAL2 at 37 °C for 30 min. The reaction gave **12** in ~70% yield (Figure 3D). Notably, the free ^α^N-amino group of the N-terminal Gly residue in the affibody was resistant to butelase-1 at the first ligation step, confirming that the GF dipeptide motif is a relatively poor nucleophile substrate of butelase-1. Because the two PALs require only a short NXY tripeptide as the recognition tag and ligate at the Asn residue, only minimal traces are left in the modified proteins. These results show the robustness and neatness of our sequential bio-orthogonal ligation method for protein dual labeling. Detailed information on the reaction protocols and conditions is summarized in Figure S4 and Table S1.

Of note, in the C-to-N scheme, the NGI sequence formed at the first BML step was not affected significantly when conducting VML, likely because **9** - with much faster diffusion kinetics than the much larger molecule **13** - was used in a 5-fold excess to **13**. In addition, the C-terminal NGI in small peptide **9** may be more accessible than the NGI sequence in **13**. Nevertheless, it would be ideal to use an incoming nucleophile peptide sequence at the first BML step that would generate a site that is sub-optimal for recognition by VyPAL2. However, when testing peptides with an N-terminal HV or GV dipeptide motif for BML in the first step, we found these peptides to be poorer nucleophile substrates than the GI-peptides for butelase-1 recognition. Indeed, the BML reaction of the affibody protein **8** with HVGGRIK(Biotin)GA, peptide **14**, yielded only ~40% of ligation product in 2 h and 60-65% in 4 h (Figure S6). This reaction was much slower than the reaction with the GI-peptide, **11**, which was nearly complete (at least 85%) in 30 min under the same conditions. The ligation reaction of **8** with a GV-peptide, GVGGRIK(Biotin)GA **19**, was even slower, providing less than 30% in 2 h (Figure S7). Therefore, although using an NH-peptide could make the C-to-N scheme a potentially more orthogonal method, such a scheme would be significantly less efficient than the one using a GI-peptide.

We also attempted a one-pot reaction using a pair of N-terminal and C-terminal labeling peptides, **7** and **14**, which are supposedly of optimal orthogonality. However, simultaneous one-pot BML and VML reactions did not yield the desired end product (Supporting Information; Figure S5). Conducting BML and VML sequentially in one pot furnished the desired dual labeled product in good yields, albeit with a significant unwanted side reaction of inter-peptide ligation (Supporting Information; Figure S6).

### Binding affinity (K_D_) of the dual labeled affibody 12 to EGFR on A431 cells

**Figure 4:**
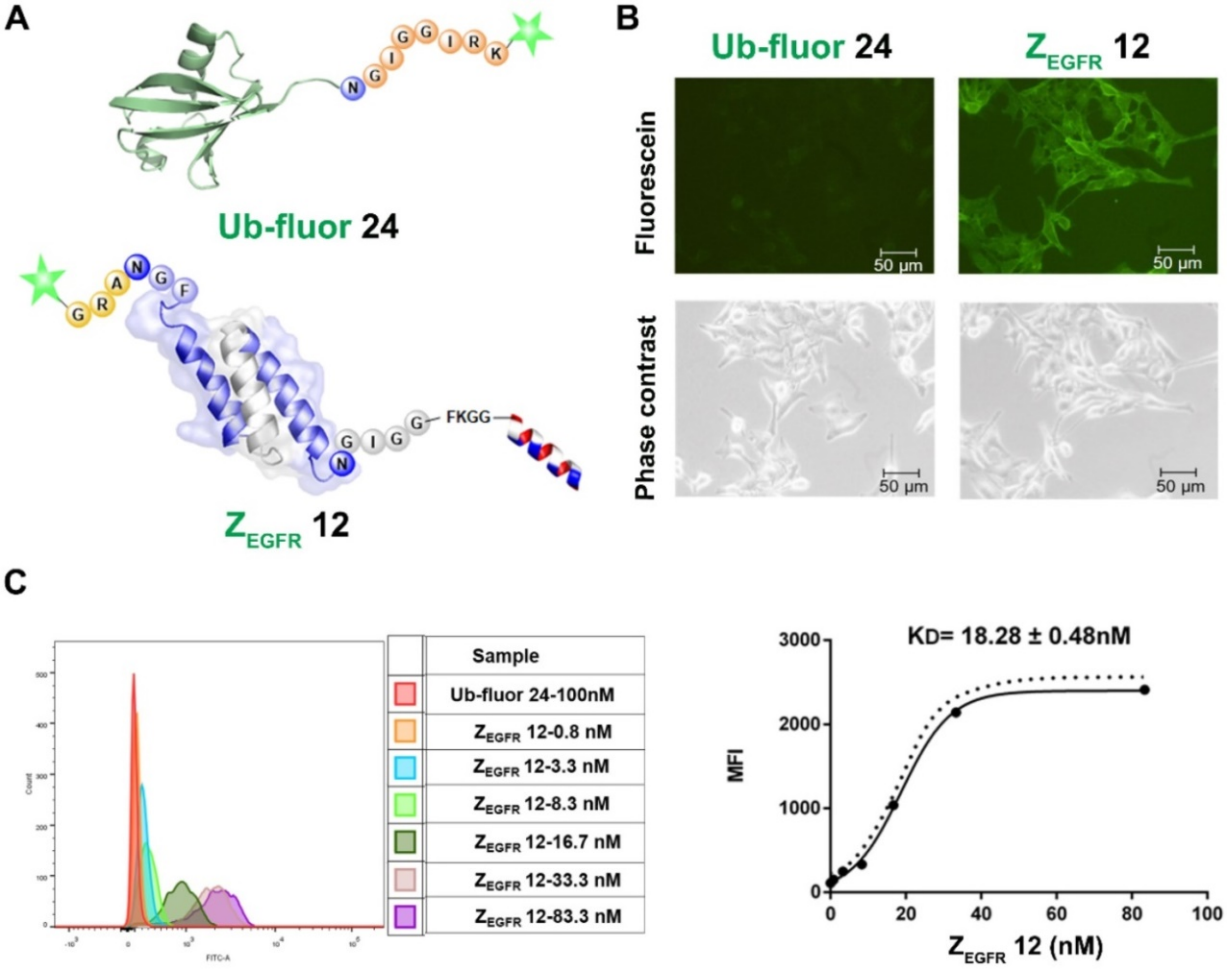
Imaging and binding study of **12** on EGFR-overexpressing A431 cells. (A) Schematic structure of ubiquitin tagged with fluorescein and dual labeled affibody **12** with fluorescein on the N-terminus and mitochondrion-lytic peptide at the C-terminal end; (B) Fluorescence microscopy analysis of **12** binding on A431 cells; (C) Determination of K_D_ of **12** in binding to A431 cells using flow cytometry.

To study the activities of the dually labeled product **12**, we analyzed its binding to EGFR-overexpressing A431 cells. The cells were treated with 100 nM of **12**, while the fluorescein-tagged ubiquitin **24**, which was prepared via BML with the fluorescent peptide **23** (Figure S8), was used as a negative control. As shown in Figure 4A, strong green fluorescence was observed in A431 cells treated with **12** (Figure 4B), whereas no fluorescence was observed in A431 cells treated with **24**. Meanwhile, FACS analysis indicated a remarkable shift in the fluorescence intensity of the **12**-treated cells in reference to ubiquitin **24**-treated cells in the control group (Figure 4C), which was consistent with the fluorescence imaging data. To determine the dissociation constant (K_D_) of **12**, these cells were treated with different concentrations of **12** and subjected to FACS analysis after 30 min of incubation. As shown in Figure 4C, treatments with different concentrations of **12** resulted in different intensity shifting. The mean fluorescent intensity was analyzed using a non-linear regression function, resulting in a K_D_ of 18.28 ± 0.48 nM.

### Cytotoxicity evaluation of the dual-labeled affibody

**Figure 5:**
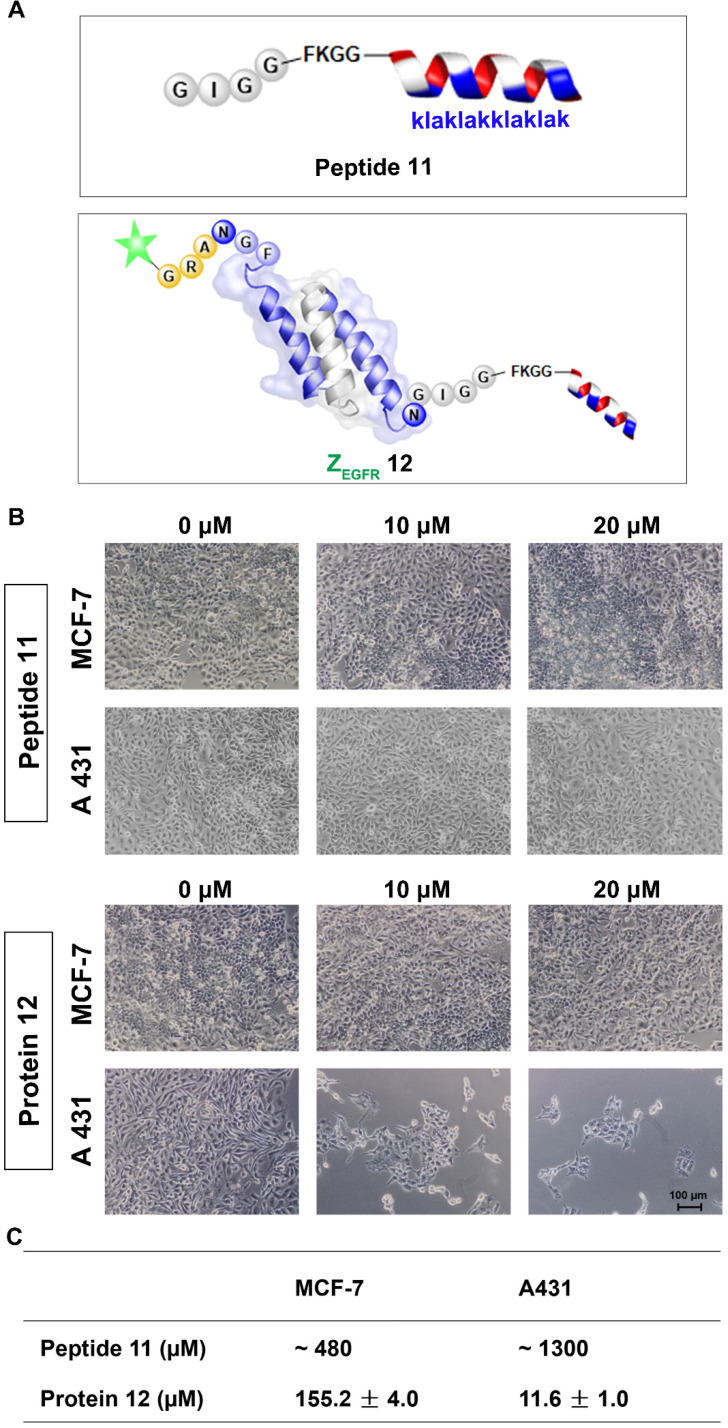
Cytotoxicity study of the dually labeled protein **12**. (A) Schematic structure of the **KLA** D-peptide **11** and dual labeled affibody **12** with fluorescein on the N-terminus and mitochondrion-lytic peptide at the C-terminal end; (B) Microscopy analysis of **11** and **12** in MCF-7 and A431. Cells were treated with phosphate buffer (as negative control) **11** or **12** for 72 h and then subjected to microscopy analysis after washing 3 times with PBS; (C) IC_50_ of **11** and **12** on MCF-7 and A431 cells. Both cells were treated with **11** or **12** for 84 h, followed by an MTT-based viability test to evaluate optical absorbance and calculate the corresponding IC_50_.

Next, we performed the MTT assay to determine whether **12** had any effects on the two cell lines, the EGFR-overexpressing A431 cells and the MCF-7 cells, which have a low EGFR expression level 54. Both cell lines were treated with **12** for 84 h and then subjected to MTT analysis. **12** exhibited significant toxicity to A431 cells with an IC_50_ of 11.61 ± 0.96 μM, whereas it showed an IC_50_ of 155.23 ± 3.99 μM for MCF-7 cells. The unconjugated peptide **11** had IC_50_ values of approximately 480 μM and 1300 μM against MCF-7 and A431 cells, respectively (Figure 5A, B and Figure S9). Owing to its poor cellular uptake 55, the low cytotoxicity of **11** itself in the two cell lines was expected 52. However, conjugating the peptide to the EGFR-targeting affibody drastically enhanced its cytotoxicity against A431 cells, likely because the affibody helped deliver the mitochondrion-lytic peptide intracellularly via EGFR-mediated endocytosis. Thus, as the internalized **12** ended up in the lysosomes, the high proteolytic activity of enzymes, such as cathepsin B, would destroy the peptide linker and even the affibody to release the **KLA** D-peptide, which, after escaping from lysosomes, would disrupt the mitochondrial membrane, leading to apoptosis.

The data clearly indicate that a protein with orthogonal N- and C-terminal recognition tags can be dually labeled by the consecutive action of two PALs with differential substrate specificities. The dually labeled affibody protein has selective imaging and cytotoxic activities. To further demonstrate the versatility of the tandem ligation scheme, we proceeded with the synthesis of a cyclic form of the affibody tagged with doxorubicin (Figure 2).

### Synthesis of a cyclic affibody-doxorubicin conjugate

**Figure 6:**
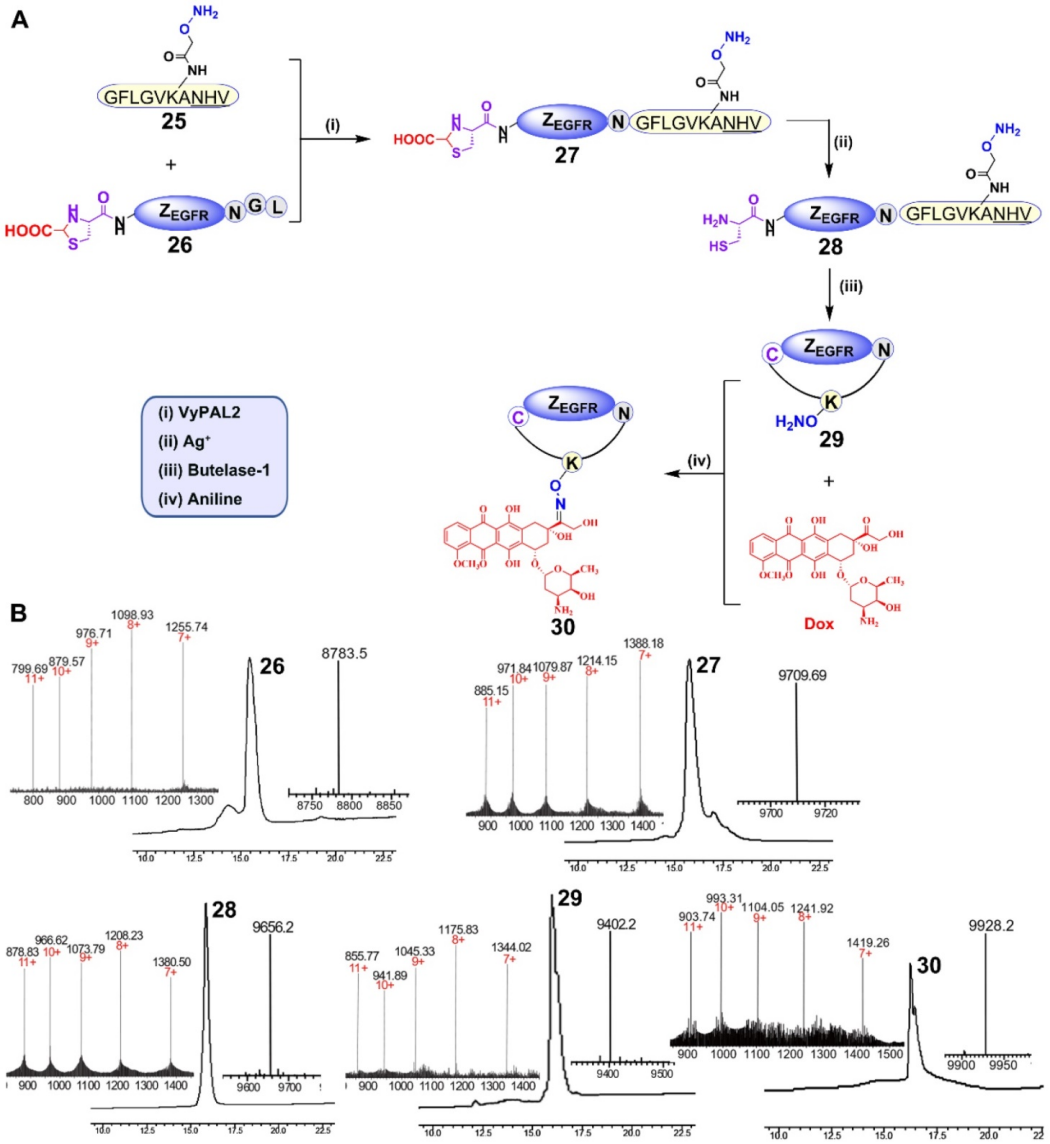
Synthesis of a cyclic affibody-drug conjugate using PAL-catalyzed orthogonal ligation and cyclization and oxime conjugation. (A) (i) Peptide **25** was tagged to the C-terminus of Z_EGFR_ **26** via VML to give **27** (90%); (ii) the N-terminal cysteine of Z_EGFR_ **27** was deprotected using silver nitrate to afford **28** (95%); (iii) Z_EGFR_ **28** was cyclized via BML to give **29** (70%); (iv) Dox was attached to Z_EGFR_ **29** via oxime conjugation to obtain final product **30** (80%). (B) HPLC and ESI characterization of purified products (**26:** calcd 8785.6, obsvd 8783.5; **27:** calcd 9708.9, obsvd 9709.7; **28:** calcd 9655.6, obsvd 9656.2;** 29:** calcd 9401.7, obsvd 9402.2;** 30:** calcd 9927.7, obsvd 9928.2).

For this purpose, peptide **25** containing an N-terminal GF dipeptide as the nucleophile substrate for VyPAL2 and a C-terminal NHV tripeptide motif at the C terminus as the electrophile substrate for butelase-1 was prepared using SPPS (Figure 6A). The aminooxy functional group in the peptide would allow for conjugation with DOX through its ketone group via oxime formation 56. The detailed synthetic route of **25** is shown in Figure S10. For obvious reasons, **8**, the affibody substrate for dual labeling, could not be used here because the intervening peptide **25** already contains the same respective nucleophile and electrophile substrates for the two PALs (Figure 6A). Therefore, affibody Z_EGFR_ **26** containing “CG-” at the N terminus and “-NGL” at the C terminus was prepared recombinantly in *E. coli*. Interestingly, ESI-MS analysis showed that the N-terminal cysteine residue of **26** was capped during protein expression, presumably as a thiazolidine moiety by the ubiquitous aldehyde metabolite glyoxylic acid in the bacterial cells, effectively blocking it from being used as a nucleophile substrate by the PAL enzymes. Thus, only the C-terminal labeling product Z_EGFR_ **27** would be generated in the first ligation step, without the possibility of cyclization or self-ligation of **26**. As expected, when VML was performed by mixing 50 μM of Z_EGFR_ **26** and 150 μM of peptide **25** with 100 nM of VyPAL2 at 37 °C for 30 min, only the C-terminal ligation product **27** was obtained, as shown clearly by HPLC and ESI-MS analysis (Figure 6B). The NHV tag in **25** or **27** was not affected, confirming its orthogonality toward VyPAL2.

To unmask the N-terminal cysteine in **27**, purified **27** (1 mM) was treated with silver nitrate (10 mM) for 30 min, followed by treatment with β-mercaptoethanol (100 mM) for 30 min. The deprotection reaction resulted in product **28**, as confirmed by HPLC and ESI-MS (Figure 6A, 6B). Butelase-mediated cyclization was performed by mixing **28** (100 μM) with 50 nM butelase-1 for 30 min at 37 °C. Cyclic product **29** was characterized by HPLC and ESI-MS (Figure 6B). The aminooxy functional group in Z_EGFR_ **29** can react with the doxorubicin ketone group via Schiff's base formation. Therefore, the oxime ligation reaction was carried out by mixing 100 μM of Z_EGFR_ **29** and 1 mM of Dox in the presence of 10 mM of aniline as the catalyst 57 at pH 6 and 37 °C overnight. The reaction gave rise to final product **30**, as characterized by HPLC and ESI-MS (Figure 6B).

### Cell imaging and cytotoxic study of the synthesized cyclic affibody-doxorubicin conjugate 30

**Figure 7:**
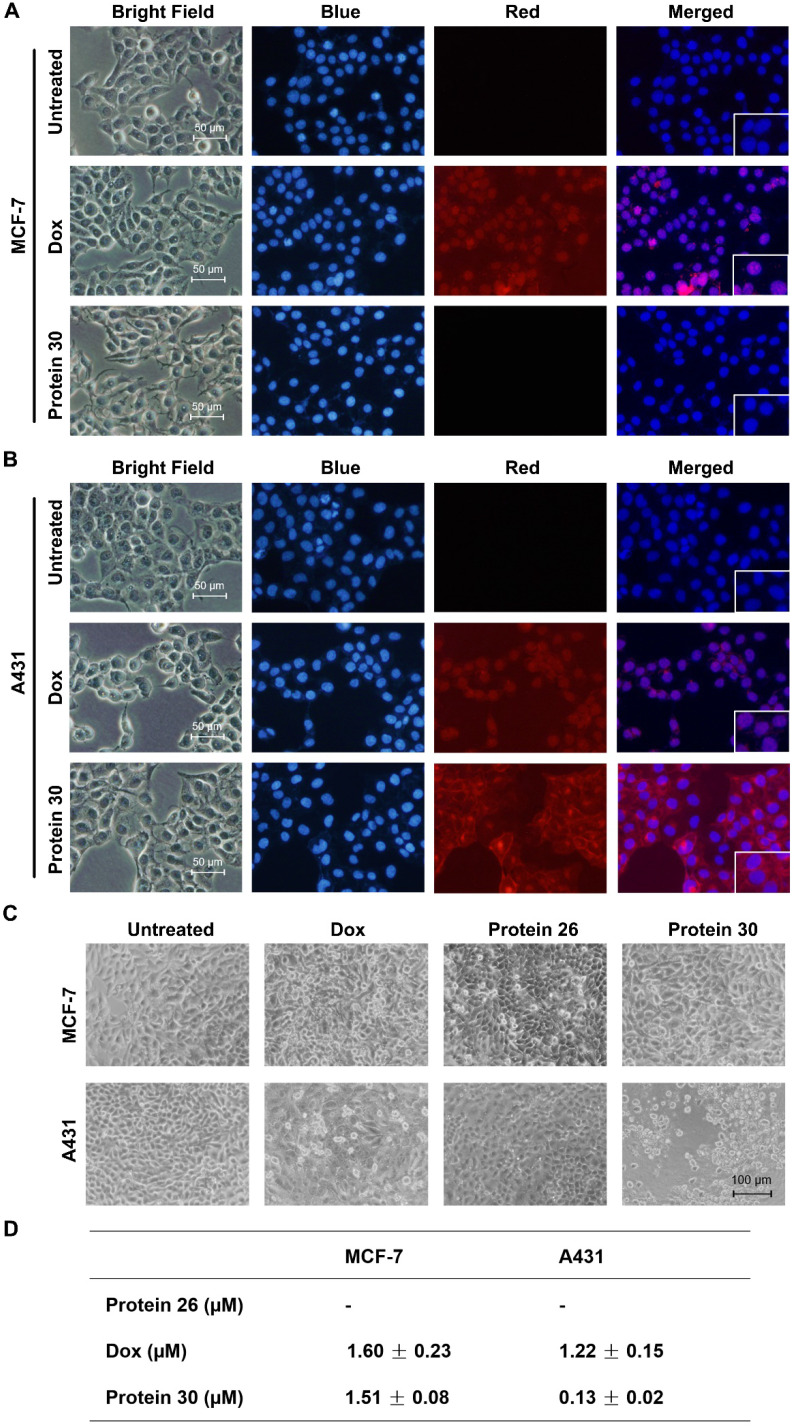
Cell imaging and cytotoxicity study of the cyclic affibody-Dox conjugate **30**. (A) Fluorescent microscopy analysis of MCF-7 cells after treatment with **30**, DOX, and blank at room temperature for 30 min. (B) Fluorescent microscopy analysis of A431 cells after treatment with **30**, DOX, and blank at room temperature for 30 min. For cell staining experiments in (A) and (B), the nucleus was stained with 700 nM of DAPI; 10 µM DOX and 2 µM **30** were used. Scale bar, 50 µm. (C) Cytotoxicity assay of the cPDC **30**. Microscopy analysis of cells treated with **30**. MCF-7 and A431 cells were treated with 0.2 µM of different molecules: DOX, unconjugated affibody **26**, and **30** for 96 h. Scale bar, 100 µm. (D) Cytotoxic IC_50_ of different compounds against MCF-7 and A431 cells. **30** exhibited a ~10-fold enhanced toxicity on the EGFR-overexpressing A431 cell line compared to doxorubicin.

Fluorescence imaging, microscopy analysis, and MTT assay were performed to determine the binding and inhibitory effects of cPDC **30** on the MCF-7 and A431 cell lines. The intrinsic fluorescence of doxorubicin serves as an imaging tool to visualize the binding of **30** to the cells (Figure 7A, B and Figure S11). As shown in the figures, only the EGFR-overexpressing A431 cells were positively stained after 30 min of treatment with **30**. The same treatment did not yield any staining of EGFR-negative MCF-7 cells. On the other hand, both cell lines were stained by free doxorubicin, which is not surprising as it can enter cells and bind to nuclear DNA. In the cytotoxicity experiments, both cell lines were treated with 0.2 μM of unconjugated affibody **26**, doxorubicin, and **30** for 96 h and subjected to microscopy analysis. At this concentration, **30** exhibited substantial cytotoxic effects on A431 cells, with smaller or no effects observed in the other control settings (Figure 7C). Next, an MTT assay was conducted to determine the IC_50_. Unconjugated affibody **26**, DOX, and cPDC **30** were added at varying concentrations to MCF-7 and A431 cells for 96 h. As shown in Figure 5D, **30** showed significantly higher toxicity with an IC_50_ of 0.13 ± 0.02 μM to A431 cells than to MCF-7 cells (IC_50_ of 1.51 ± 0.08 μM). The affibody itself had a low level of cytotoxicity, even at very high concentrations, which is consistent with previously published results 58. The unconjugated DOX showed lower cytotoxicity in terms of IC_50_ to A431 cells compared to **30**, likely due to a lack of receptor-mediated enrichment of the compound in the cells. The measured IC_50_ of DOX in MCF-7 and A431 was 1.60 ± 0.23 μM and 1.22 ± 0.15 μM, respectively (Figure 7D and Figure S12). The enhanced toxicity of the cycloaffibody-DOX conjugate **30** was likely due to the fast enrichment of the conjugate via receptor-mediated endocytosis, which led to the uptake of the conjugate through the endosomal pathway and delivered it to the lysosome. The acidic milieu in this organelle would help cleave the oxime linkage to release DOX 56. Owing to its hydrophobic properties, doxorubicin could easily escape from the lysosome to bind to nuclear DNA, leading to apoptotic cell death.

## Conclusion

Butelase-1 and VyPAL2 are PAL enzymes that recognize short peptide tags for ligation reactions. We exploited the different substrate specificities of these two PALs to develop a new method for the bio-orthogonal dual modification of proteins under mild aqueous conditions at a near neutral pH. We have used this novel bio-orthogonal method to prepare a dual-labeled affibody as a selective imaging and cytotoxic agent for the cancer cells. Our results have shown that our bio-orthogonal ligation scheme is bi-directional, as it can be executed in both the N-to-C and C-to-N directions, enabling the synthesis of the affibody conjugate **12**. Furthermore, the scheme was extended to the preparation of a cyclic affibody conjugated with the cytotoxic compound doxorubicin. Unlike the hydrophobic free doxorubicin, which is poorly soluble in water, the prepared cycloaffibody-DOX conjugate **30** showed excellent water solubility. Such a conjugate is also expected to have lower cardiotoxicity than free doxorubicin. A backbone-cyclized protein is known to have increased thermal, chemical, and proteolytic stability. As demonstrated by our data, the prepared linear and cyclic affibody conjugates **12** and **30** showed uncompromised high binding affinity and enhanced cytotoxicity toward EGFR-overexpressing A431 cells. These findings indicate that PALs show promise as a tool for the precision biomanufacturing of complex bioconjugates with multiple functionalities and unusual structures, as well as the use of these ligases for the functionalization of protein nanoparticles. Therefore, the methodologies described in this study may pave the way for the development of next-generation protein-based theranostics for the diagnosis, prevention, and treatment of disease in humans.

## Figures and Tables

**Table 1:** Kinetics of VyPAL2- and butelase-1-mediated intermolecular ligation

Electrophile substrate	Nucleophile substrate	Enzyme	*k*_cat_ [s^-1^]	*K*_m_ [μM]	*k*_cat_/*K*_m_ [M^-1^s^-1^]
Ac-**KKLAVINHV 1**	GIGGIKA **2**	VyPAL2	0.17 ± 0.01	182 ± 6	932 ± 32
		Butelase-1	1.47 ± 0.04	85 ± 3	17265 ± 465
YKANGL **4**	**GFGGIKA 5**	VyPAL2	8.29 ± 0.48	424 ± 26	19559 ± 164
		Butelase-1	1.55 ± 0.01	365 ± 3	4256 ± 52
Ac-**KKLAVINGF 7**	GIGGIKA **2**	VyPAL2	1.10 ± 0.03	155 ± 15	7219 ± 513
		Butelase-1	0.46 ± 0.04	175 ± 1	2652 ± 218

Note: Kinetic parameters of each reaction are for the substrate with the sequence in bold.

## Abbreviations

PALs: peptidyl asparaginyl ligases
POI: protein of interest
AEP: asparaginyl endopeptidase
BML: butelase-mediated ligation
VML: VyPAL-mediated ligation
NHV: Asn-His-Val tripeptide
NGF: Asn-Gly-Phe tripeptide
Vy: *Viola Yedoensis*
PBS: phosphate saline buffer
Ni-NTA: nitrilotriacetic acid-nickel
DMEM: Dulbecco's Modified Eagle Medium
FBS: fetal bovine serum
EDTA: Ethylenediaminetetraacetic acid
MTT: 3-(4,5-Dimethylthiazol-2-yl)-2,5-Diphenyltetrazolium Bromide
DAPI: 4', 6-Diamidino-2-Phenylindole, Dihydrochloride
MBHA: 4-Methylbenzhydrylamine
Fmoc: Fluorenylmethyloxycarbonyl
Boc: tert-butyloxycarbonyl
TFA: Trifluoroacetic acid
HPLC: High-performance liquid chromatography
TIS: Triisopropylsilane
ESI-MS: electrospray ionization mass spectrometry
K_D_: equilibrium dissociation constant
DMF: dimethylformamide
DCM: Dichloromethane
PyBOP: benzotriazol-1-yl-oxytripyrrolidinophosphonium hexafluorophosphate
DIPEA: N,N-Diisopropylethylamine
cPDC: cycloprotein-drug conjugate
IPTG: Isopropyl β-D-1-thiogalactopyranoside
EGFR: epidermal growth factor receptor
K_D_: dissociation constant
DOX: doxorubicin
SPPS: solid phase peptide synthesis
