# Supplementary material for: Engineering protein theranostics using bio-orthogonal asparaginyl peptide ligases
Source: Theranostics. 2021 Apr 3;11(12):5863–75. doi: 10.7150/thno.53615 (PMC8058723; doi:10.7150/thno.53615)
Supplement: Supplementary file 1 — Supplementary figures and tables. [file thnov11p5863s1.pdf]

# Supporting Information

## Engineering protein theranostics using bio-orthogonal asparaginyl peptide ligases

Zhen Wang,† Dingpeng Zhang,† Xinya Hemu, Side Hu, Janet To, Xiaohong Zhang, Julien Lescar, James P. Tam* and Chuan-Fa Liu*

School of Biological Science, Nanyang Technological University, 60 Nanyang Drive, Singapore 637551

## Table of Contents

**1. General information ........................................................ 2**

**2. Detailed experimental procedures ........................................................ 4**

**a) Kinetics of VyPAL2 and butelase-1 toward the NHV-ending acyl peptide substrate (peptide 1) and the GF-starting nucleophile peptide substrate (peptide 5)**

**b) Characterization of a by-product in the N-to-C tandem ligation using VyPAL2 and butelase-1**

**c) Summary of materials used in the tandem ligation reactions**

**d) One-pot reaction**

**e) BML for labeling of ubiquitin containing C-ter NHV**

**f) $IC_{50}$ determination using MTT cell viability assay**

**3. Mass spectra and HPLC profiles ........................................................ 18**

## 1. General information

**Protein amino acid sequences**

The optimized DNA sequences of DARPin and $Z_{EGFR}$ were synthesized by GenScript. The amino acid sequences were as follows:

$Z_{EGFR}$ **8**

GFGSSLQVDNKFNKEMWAAWEEIRNLPNLNGWQMTAFIASLVDDPSQSANLLAEAKKLNDA
QAPKVDGSGSNHVHHHHHH

Ubiquitin **22** protein sequence

GGSGSGSQIFVKTLTGKTITLEVEPSDTIENVKAKIQDKEGIPPDQQRLIFAGKQLEDGRTLSDY
NIQKESTLHLVLR LRGGNHVHHHHHH

$Z_{EGFR}$ **26**

CGSSHHHHHHLQVDNKFNKEMWAAWEEIRNLPNLNGWQMTAFIASLVDDPSQSANLLAEAK
KLNDAQAPKVDGSGSNGL

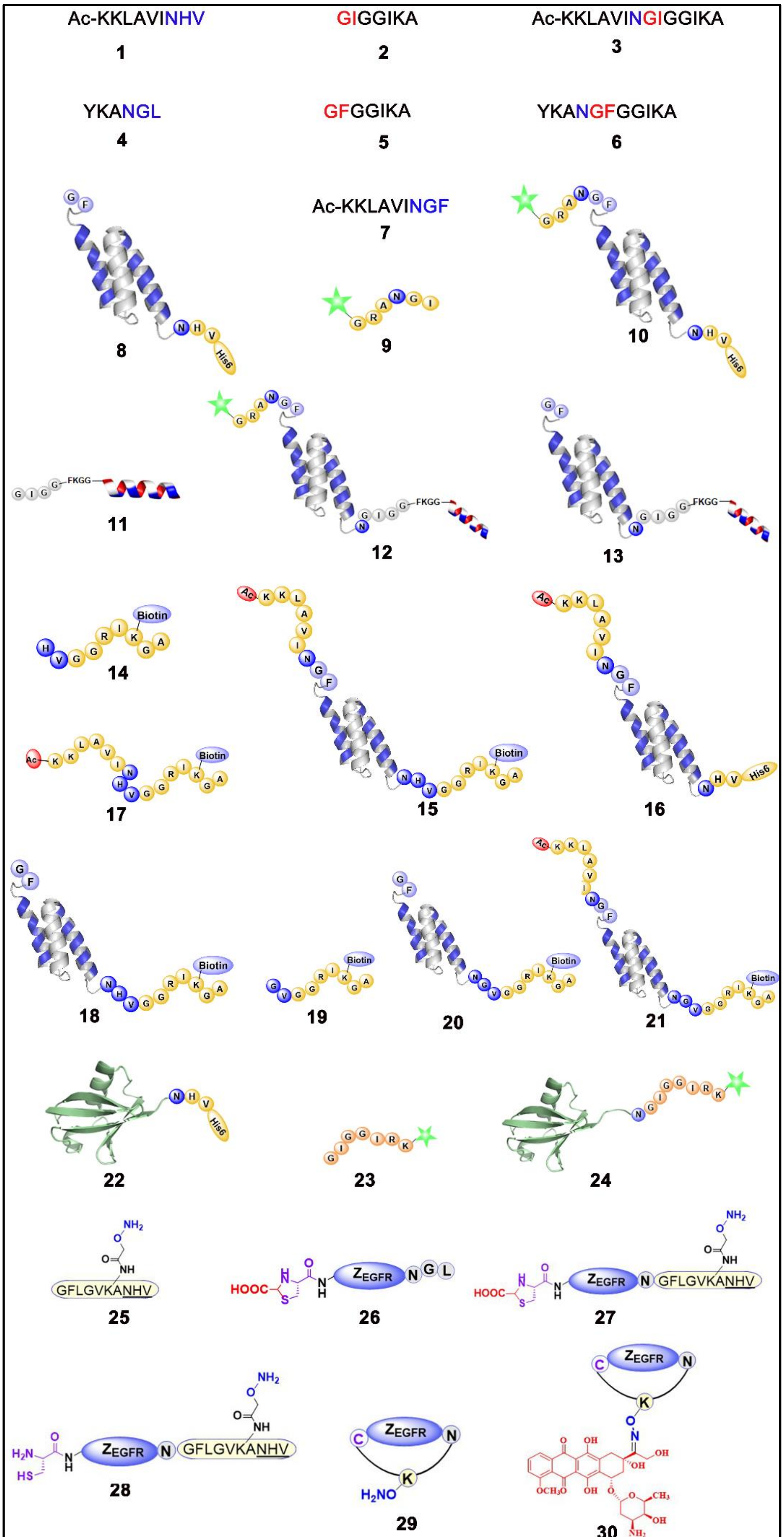
Ac-KKLAVINHV
1
GIGGIKA
2
Ac-KKLAVINGIGGIKA
3
YKANGL
4
GFGGIKA
5
YKANGFGGIKA
6
Ac-KKLAVINGF
7
8
9
10
11
12
13
14
15
16
17
18
19
20
21
22
23
24
GFLGVKANHV
25
$Z_{EGFR}$
26
27
28
29
30

**Figure S1.** Numbering and illustrative structures of compounds used in this study. The green star indicates 5(6)-carboxyfluorescein coupled through its carboxyl group to the side-chain amine of a lysine residue or to the N-terminal amine.

## 2. Detailed experimental procedures

### a. Kinetics of VyPAL2 and butelase-1 toward the NHV-ending acyl peptide substrate (peptide 1) and the GF-starting nucleophile peptide substrate (peptide 5)

To demonstrate and quantify the differential specificities of two PAL enzymes, we evaluated the kinetics of peptide ligations between peptides **1** (Ac-KKLAVINHV) and **2** (GIGGIKA) using butelase-1 and VyPAL2, respectively (Figure S6A). The ligation reaction between **1** and **2** yielded peptide **3** Ac-KKLAVINGIGGIKA (ESI-MS: 1423.09 obsv, 1422.28 calc). The reactions were performed by adding different concentrations of Ac-KKLAVINHV **1** (50, 100, 200, 400, 600, and 800 μM) with nucleophile **2** GIGGIKA (kept at 1 mM). Then, 40 nM of VyPAL2 or 10 nM of butelase-1 was added to the above reaction for 30 min at pH 6.5. Aliquots of the reaction mixtures were analyzed by HPLC. The results showed that the catalytic efficiency of butelase-1 was ~18-fold that of VyPAL2 toward the "NHV" substrate peptide **1**. Similarly, the catalytic activities of VyPAL2 and butelase-1 towards the GF-nucleophile peptide GFGGIKA **5** were measured. We performed the reactions using different concentrations of **5**, (50, 100, 200, 600, and 800 μM) with the acyl-side substrate YKAINGL **4** (kept at 1.5 mM). butelase-1 and VyPAL2 were added at 50 nM and 13 nM, respectively. The ligation reaction between peptides **4** and **5** yielded peptide **6** YKAINGFGGIKA (ESI-MS: 1124.91 obsv, 1124.15 calc). We found that VyPAL2 was ~5 times more efficient than butelase-1 toward the nucleophile substrate GFGGIKA **5**. Therefore, our results confirm earlier observations that, while the NHV sequence is an excellent substrate of butelase-1, it is a poor substrate of VyPAL2. Furthermore, these results indicate that the N-terminal GF dipeptide is a good substrate of VyPAL2, but not a very good substance of butelase-1. Similarly, the kinetics of butelase-1 and VyPAL2 towards another acyl donor substrate, peptide **7** (Ac-KKLAVINGF), in ligation with GI-peptide **2** were also determined. The reactions were performed using a varying concentration of **7** and a constant concentration of **2** in the presence of butelase-1 (100 nM) or VyPAL2 (50 nM). We found that the NGF motif was 6.5-fold less active than the NHV motif in butelase-mediated ligation and that VyPAL2 was about 2.6-fold more efficient than butelase-1 towards peptide **7**.

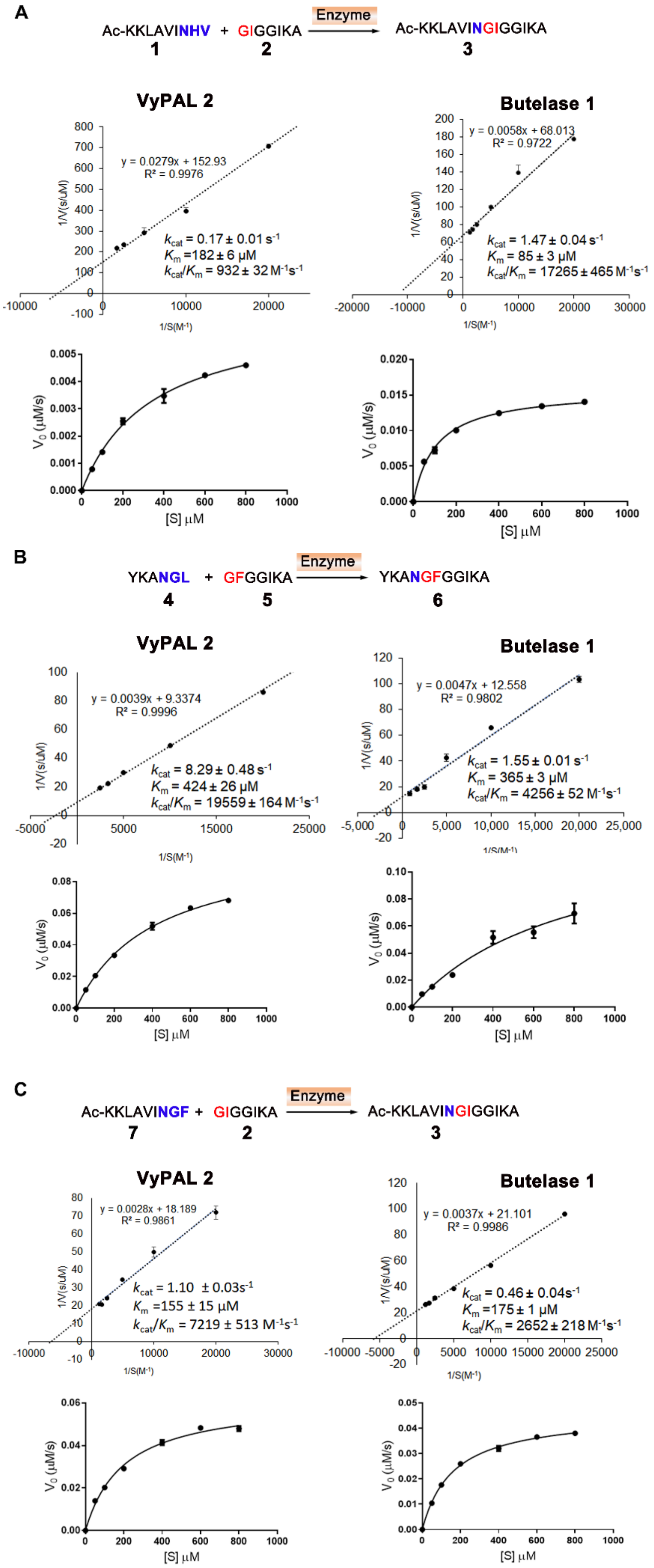
A
Enzyme
Ac-KKLAVINHV + GIGGIKA → Ac-KKLAVINGIGGIKA
1
2
3
VyPAL 2
y = 0.0279x + 152.93
R² = 0.9976
kcat = 0.17 ± 0.01 s-1
Km =182 ± 6 µM
kcat/Km = 932 ± 32 M-1s-1
1/V(s/uM)
1/S(M-1)
Butelase 1
y = 0.0058x + 68.013
R² = 0.9722
kcat = 1.47 ± 0.04 s-1
Km = 85 ± 3 µM
kcat/Km = 17265 ± 465 M-1s-1
V0 (µM/s)
[S] µM
B
Enzyme
YKANGL + GFGGIKA → YKANGFGGIKA
4
5
6
VyPAL 2
y = 0.0039x + 9.3374
R² = 0.9996
kcat = 8.29 ± 0.48 s-1
Km = 424 ± 26 µM
kcat/Km = 19559 ± 164 M-1s-1
Butelase 1
y = 0.0047x + 12.558
R² = 0.9802
kcat = 1.55 ± 0.01 s-1
Km = 365 ± 3 µM
kcat/Km = 4256 ± 52 M-1s-1
C
Enzyme
Ac-KKLAVINGF + GIGGIKA → Ac-KKLAVINGIGGIKA
7
2
3
VyPAL 2
y = 0.0028x + 18.189
R² = 0.9861
kcat = 1.10 ± 0.03s-1
Km =155 ± 15 µM
kcat/Km = 7219 ± 513 M-1s-1
Butelase 1
y = 0.0037x + 21.101
R² = 0.9986
kcat = 0.46 ± 0.04s-1
Km =175 ± 1 µM
kcat/Km = 2652 ± 218 M-1s-1

**Figure S2.** Determination of kinetic parameters of VyPAL2- and butelase-1-catalyzed intermolecular ligations by Michaelis-Menten and Lineweaver-Burk plotting. A) Peptide **1** containing the C-ter NHV sequence was used at varying concentrations (50–800 µM) to react with peptide **2** at a constant concentration (1 mM); B) Peptide **4** at a constant concentration (1.5 mM) was reacted with the GF-peptide **5** at varying concentrations (50–800 µM). C) Peptide **2** at a constant concentration (1.5 mM) was reacted with the -NGF peptide **7** at varying concentrations (50–800 µM). The ligation product **3** or **6** was confirmed by ESI-MS. The reaction rates were calculated from the consumption of the limiting substrate **1**, **5**, or **7**. Initial rates ($V_0$) at different concentrations of the limiting substrate were used for Michaelis-Menten curve plotting. For kinetic parameter calculation, a Lineweaver-Burk plot was used for the analysis.

## b. Characterization of a by-product in the N-to-C tandem ligation using VyPAL2 and butelase-1

Reaction procedures, conditions, and product characterization data of tandem ligation experiments are found in the main text. The analysis is of a minor by-product formed in the second step of the N-to-C tandem ligation scheme, which resulted from the cleavage of the N-G bond catalyzed by butelase-1.

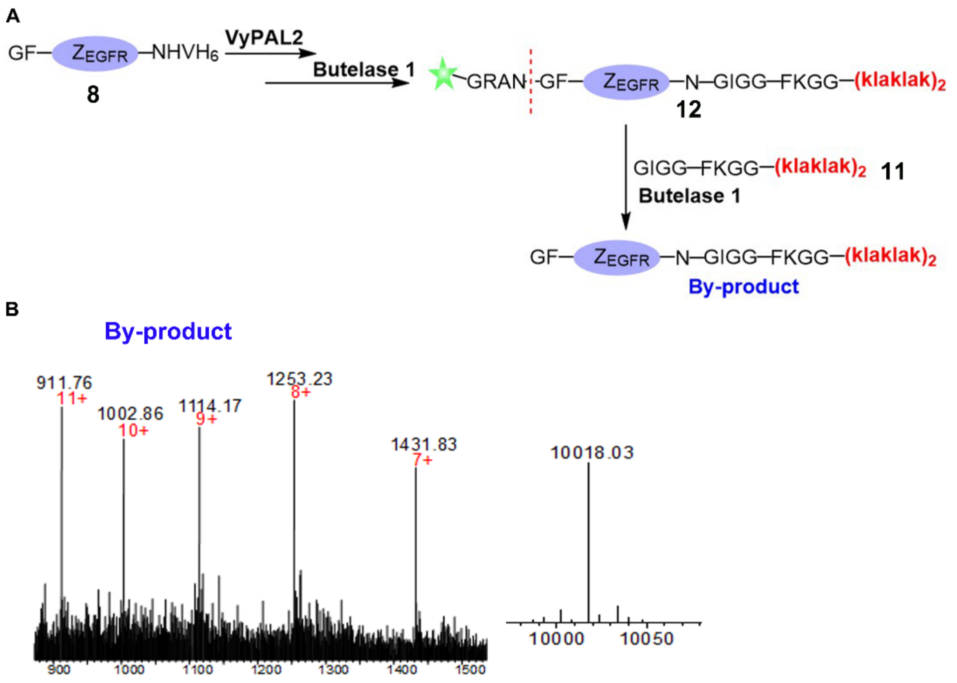


**Figure S3.** Characterization of a minor by-product in the 2nd step of the N-to-C tandem ligation. A) Schematic of the by-product formation resulting from butelase1-mediated nucleophilic attack by peptide **11** to the N-G peptide bond in $Z_{EGFR}$ **12**; B) ESI-MS characterization of the isolated by-product (calculated mass: 10018; observed mass: 10018).

## c. Summary of materials used in the tandem ligation reactions

GF-$\mathbf{Z}_{EGFR}$-NHV protein **8** was prepared by recombinant expression and used for tandem ligation. The tandem ligation scheme was carried out in both the N-to-C and C-to-N directions (Figure S4). In the N-to-C ligation scheme, VyPAL2 was used in the first step to label **8** with the fluorescein-peptide **9** to the N-terminus. Butelase-1 was used in the second step to label the protein with the mitochondrial lytic peptide **11** to the C-terminus. In the C-to-N ligation scheme, the same materials were used as in N-to-C ligation, but the reactions were carried out in the reverse order. The amount of material used is

summarized in Table **S1**. The reactions were performed at 37 °C for 20–30 min.

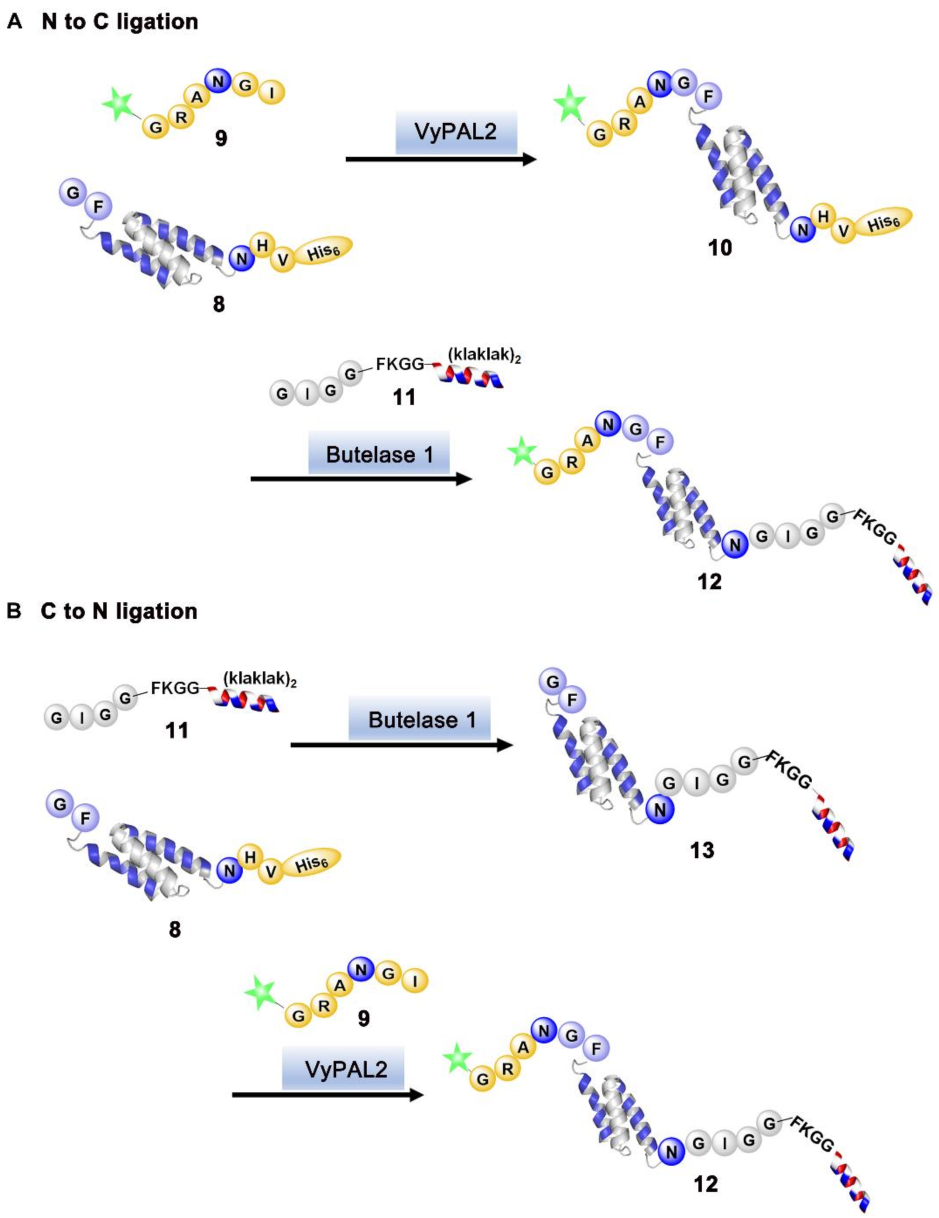


**Figure S4.** Tandem ligation protocol. A) N-to-C tandem ligation. Fluorescein-peptide **9** was first ligated to the N terminus of $Z_{EGFR}$ **9** via VML to give **11**, which, after purification, was then ligated with peptide **11** at C terminus via BML to give **12**; B) C-to-N tandem ligation. Mitochondrion-lytic peptide **11** was conjugated at C terminus of $Z_{EGFR}$ **9** to give **13** via BML and then the fluorescein-peptide **9** was ligated to the N terminus of purified **13** to produce **12**. The purified final product **12** was refolded before use in the assays.

**Table S1.** Summary of material used in the tandem ligation reactions.

| | **N-to-C** | | | | **C-to-N** | | | |
|---|---|---|---|---|---|---|---|---|
| | **Step 1** | | **Step 2** | | **Step 1** | | **Step 2** | |
| **Protein** | Protein **8** | 50 μM | Protein **10** | 50 μM | Protein **8** | 50 μM | Protein **13** | 50 μM |
| **Peptide** | Peptide **9** | 250 μM | Peptide **11** | 250 μM | Peptide **11** | 250 μM | Peptide **9** | 250 μM |
| **Enzyme** | VyPAL2 | 150 nM | Butelase 1 | 100 nM | Butelase 1 | 100 nM | VyPAL2 | 150 nM |

**d. One-pot reaction**

First, we attempted one-pot reactions with simultaneous VML and BML. To ensure good orthogonality, peptide **7** (which has a C-terminal NGF tripeptide motif) and peptide **14** (with an N-terminal HV dipeptide motif) were chosen for N- and C-terminal labeling, respectively. We performed the reaction using 50 μM of affibody **8**, 250 μM of peptide **14**, and 250 μM of peptide **7** in the presence of 250 nM of butelase-1 and 160 nM of VyPAL2. The protein and peptide starting materials were pre-mixed, and then a mixture of butease-1 and VyPAL2 was added. The reaction mixture was incubated at 37 °C for 45 min. As a result, the predominant reaction was found to be inter-peptide ligation (data not shown). While a significant amount of the N-terminal labeling product resulting from VML was formed, the desired end-product was not detected (data not shown). Next, the amount of peptide **7** (500 μM; peptide **14** was kept at 250 μM; ratio of peptide **14**: peptide **7** = 1:2) in the reaction was increased (Figure S5). After 45 min of reaction, only the intermediate product **16** was found, and no end-product **15** was formed, or an amount too small for characterization. Again, a large amount of inter-peptide ligation product **17** was formed (Figure S5B). VML for N-terminal protein ligation appeared to be much faster than BML. Although adding more butelase-1 would help accelerate C-terminal ligation, this would also increase the rate of inter-peptide ligation. Similarly, the amount of VyPAL2 could be decreased, which may help to balance the two ligations; however, this will cause the overall reaction to be too slow to be practically viable. However, inter-peptide ligation is not avoidable. Therefore, a one-pot reaction of simultaneous VML and BML is not recommended.

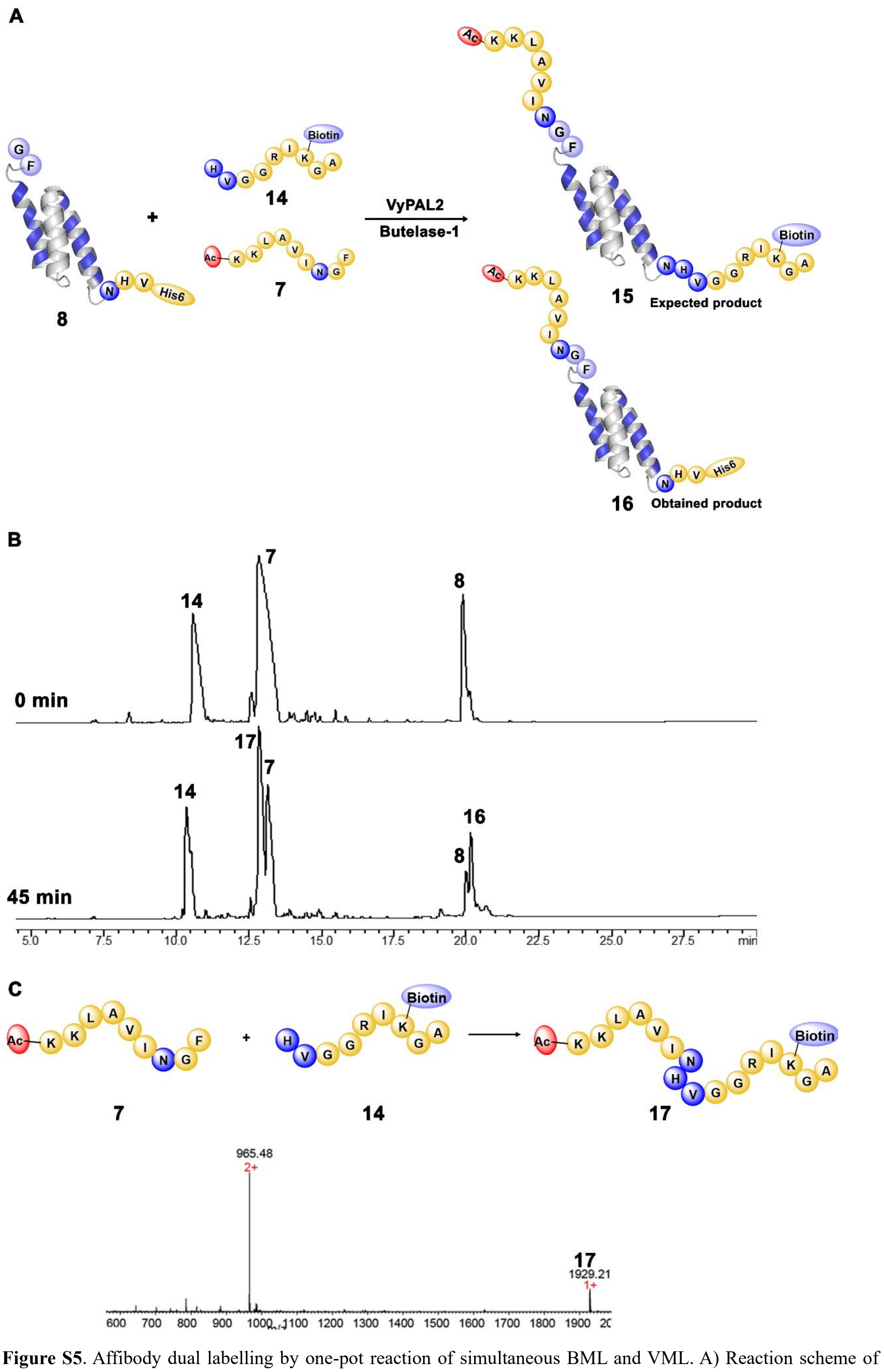


**Figure S5**. Affibody dual labelling by one-pot reaction of simultaneous BML and VML. A) Reaction scheme of simultaneous one-pot ligations. B) HPLC monitoring of the simultaneous one-pot reaction. Upper panel: mixture of peptide 14 (250 μM), **7** (500 μM), and protein **8** (50 μM) before the addition of the enzymes; Lower panel: the

reaction mixture at 45 min, after simultaneous addition of butelase-1 (250 nM) and VyPAL2 (160 nM). C) Formation of the inter-peptide ligation product **17** resulting from **7** reacting with **14** in the reaction mixture containing the two PAL enzymes (calcd. 1927.4, obsvd. 1929.2).

Next, we performed sequential ligation reactions in one pot. When the sequence of the reactions was VML first and BML second, a very small amount of the end-product was obtained (data not shown). This is due to the fact that, firstly, a large amount of peptide **7** was present, which could react with the subsequently added peptide **14** (which was intended for protein C-terminal labeling via BML), and, secondly, the N-terminal labeled product **16** could also react with **14** presumably through catalysis by VyPAL2, causing transpeptidation at the newly formed N-G bond in **16** to yield **8**. VML appeared to be much more efficient than BML in this reaction setting. Together, this would cause the unproductive consumption of peptide **14**. Therefore, sequential one-pot VML-BML reactions were not useful. Next, we performed one-pot sequential ligations in the reverse order. To this end, first butelase-1 was added to the reaction mixture containing **8** (50 μM) and HV-peptide **14** (250 μM). After incubation (Figure S6), peptide **7** (50 μM) and VyPAL2 were added. As seen from Figure S6B, the butelase-mediated ligation of the affibody protein **8** with HVGGRIK(Biotin)GA **14** yielded ~40% of ligation product **18** in 2 h and 60-65% in 4 h (Figure S6B). We found that both the C-terminal labeling product **18** and unreacted **8** from the first step were completely reacted with **7** and cleanly converted to their respective products **15** and **16** within 45 min. However, significant side reactions involving ligation between the two small peptides **7** and **14** occurred, which gave a large quantity of the inter-peptide ligation product **17** that eluted closely with peptide **7** (**17** slightly before **7**, but not resolved in the profiles shown in Figure S6B).

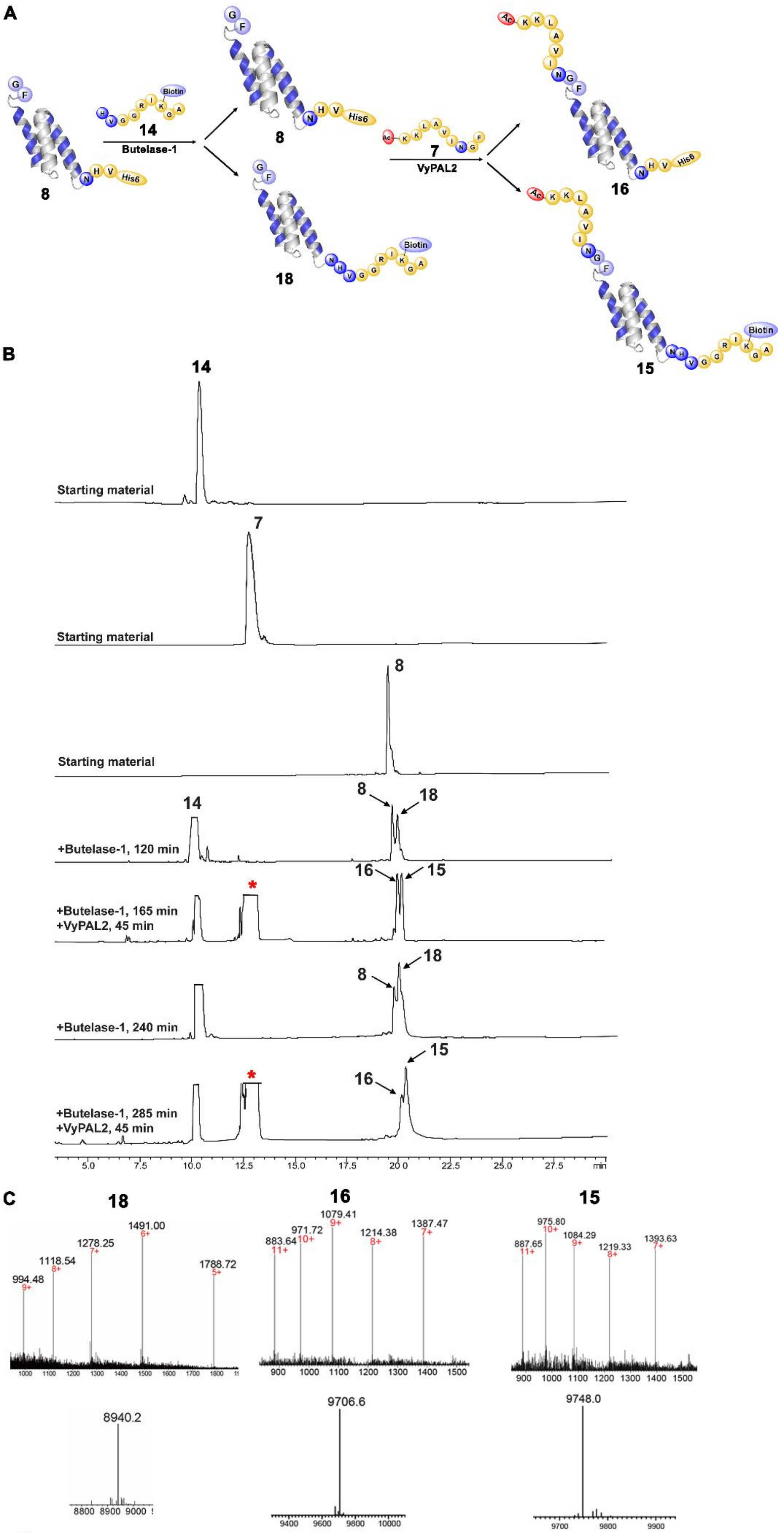


*Peak containing both 7 and inter-peptide ligation product 17 (slightly before 7)

**Figure S6.** Dual labelling of affibody **8** with an HV-peptide by butelase-1 (C-terminal labelling) and an –NGF peptide by VyPAL2 (N-terminal labelling). A) Schematic illustration of affibody dual labelling by sequential BML and VML in one pot. B) HPLC analysis. The first three HPLC profiles correspond to the starting materials: peptide **14**, peptide **7**, and protein **8**, respectively. For dual labelling of the affibody, butelase-1 was first added to the reaction mixture containing **8** and HV-peptide **14**. At 120 min, an aliquot of the reaction mixture was taken out for HPLC analysis. At the same time, the reaction mixture was divided to two halves. Peptide **7** and VyPAL2 were added to the first half of the reaction mixture. After 45 min, an aliquot of the reaction was analyzed by HPLC. The second half was allowed to continue the reaction under BML for another 120 min, at which time peptide **7** and VyPAL2 were added. After 45 min, an aliquot of the reaction mixture was analyzed by HPLC. **16** was formed by VML of the unreacted starting material **8** (left-over from the BML step) with peptide **7**. **18** was formed from BML of **8** with **14**. **15** is the desired final product formed by VML of **18** with **7**; C) Characterization of **18**, **16**, and **15** by ESI-MS (**18**: calcd. 8937.8, obsvd. 8940.2; **16**: calcd. 9705.8, obsvd. 9706.6; **15**: calcd. 9753.8, obsvd. 9748.0).

Using a GV-peptide such as GVGGRIK(Biotin)GA, **19**, was also conceivable for a more orthogonal ligation scheme in one pot. However, the BML reaction with **19** was even slower than that with **14**. Despite a large excess of **19** (400 μM) to **8** (50 μM), the reaction yielded less than 30% of ligation product in 2 h. Therefore, because the first BML step would take a very long time to complete, the overall efficiency of using a GV-peptide as the nucleophile substrate for the sequential ligation scheme would be very low, despite the second VML step being relatively fast (Figure S7). However, it is worth noting that there was significant inter-peptide ligation when the two peptides **7** and **19** were present in the reaction mixture (Figure S7).

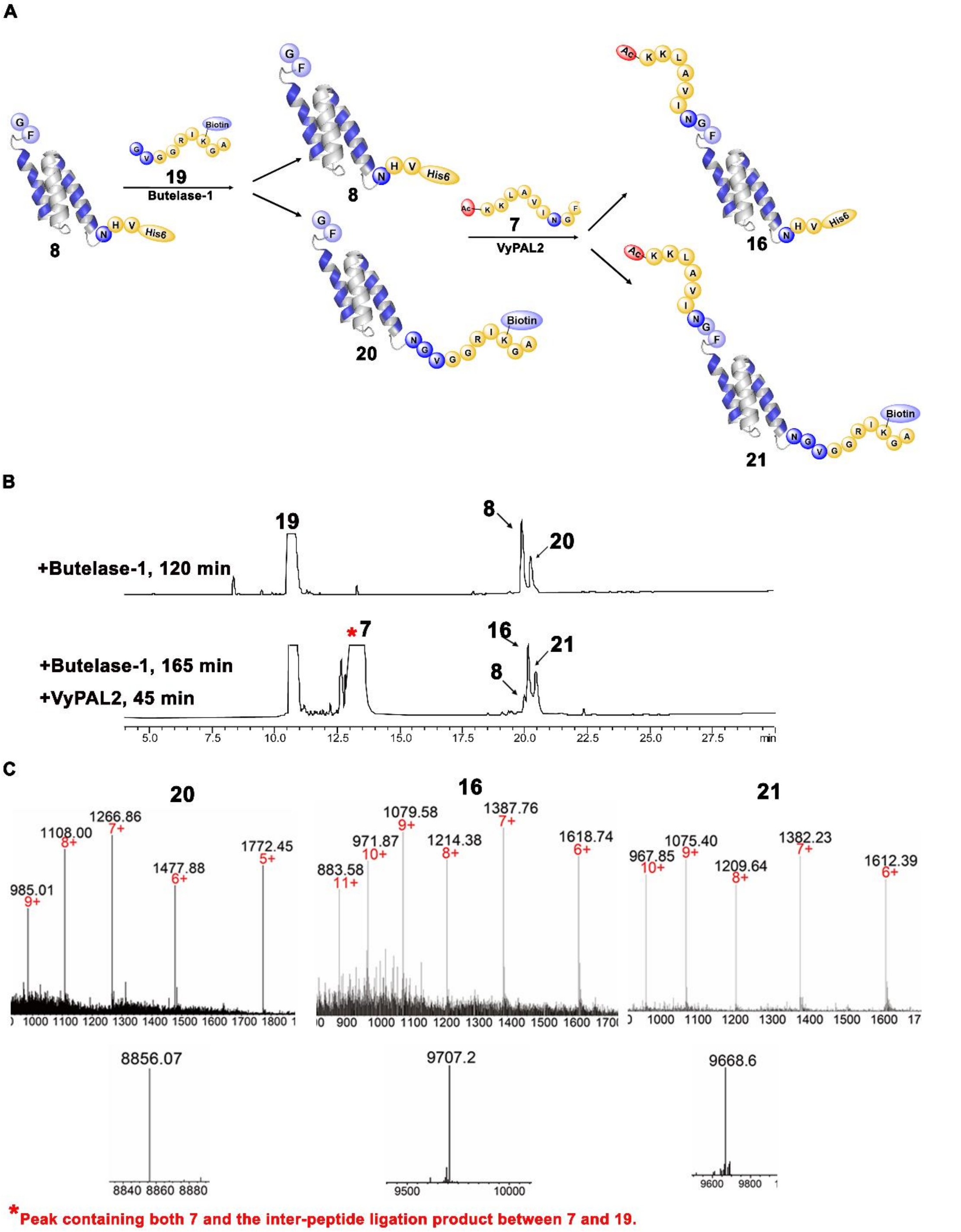


**Figure S7**. Butelase-1- and VyPAL2-mediated affibody dual labelling in one-pot reaction. A) Schematic illustration of the one-pot ligation; B) HPLC profiling of sequential one-pot ligation. Butelase-1 was first added to the reaction mixture containing affibody **8** (50 μM) and the GV-peptide **19**. After 120 min, VyPAL2 and peptide **7** were added. Upper panel: HPLC analysis of affibody **8** ligating with peptide **19** catalyzed by butelase-1 at 120 min. As shown in the figure, less than 30% of BML product **20** was formed from the first step after 120 min of reaction. Product **20** was isolated and the mass was confirmed using ESI-MS. Lower panel: HPLC profiling of the reaction mixture 45 min after addition of peptide **7** and VyPAL2. **16** was formed by VML of the unreacted starting material **8** with peptide **7**; C) Characterization of **20**, **16**, and **21** via ESI-MS (**20**: cal. 8856.5, obs. 8856.1; **16**: cal. 9706.1, obs. 9707.2; **21**: cal. 9667.9, obs. 9668.6).

Kinetic studies on the GV- and HV-peptides in comparison with the GI-peptide in BML with the NHV peptide **1** were also performed. Indeed, we found that these two nucleophile substrates were inferior to the GI-peptide in the reaction kinetics (Table S2).

**Table S2**. Kinetics of butelase-1-mediated ligation of peptide **1** with different nucleophile substrates.

| Electrophile substrate | Nucleophile substrate | $k_{cat}$ [$s^{-1}$] | $K_m$ [μM] | $k_{cat}/K_m$ [$M^{-1}s^{-1}$] |
|---|---|---|---|---|
| Ac-KKLAVINHV **1** | **GI**GGIKA **2** | 8.38 ± 0.4 | 633 ± 41 | 13253 ± 230 |
| Ac-KKLAVINHV **1** | **GV**GGRIK(Biotin)GA **19** | 2.35 ± 0.06 | 352 ± 13 | 6671 ± 128 |
| Ac-KKLAVINHV **1** | **HV**GGRIK(Biotin)GA **14** | 5.14 ± 0.8 | 519 ± 17 | 8714 ± 106 |

### e. BML for labeling of ubiquitin 22 containing C-ter NHV

Fluorescent ubiquitin **24** was prepared from Ub-NHVH$_6$ **22** and peptide **23** via butelase-mediated ligation (BML). The reaction was performed by mixing 50 μM of **22** and 250 μM of **23** with 100 nM of butelase-1 for 30 min. The product **24** was then purified by HPLC. The purified product **24** was subjected to refolding using the serial dilution method. The lyophilized powder was dissolved in 6 M guanidine HCl (pH 7, phosphate buffer) and dialyzed against decreasing concentrations of guanidine HCl buffers until pure PBS.

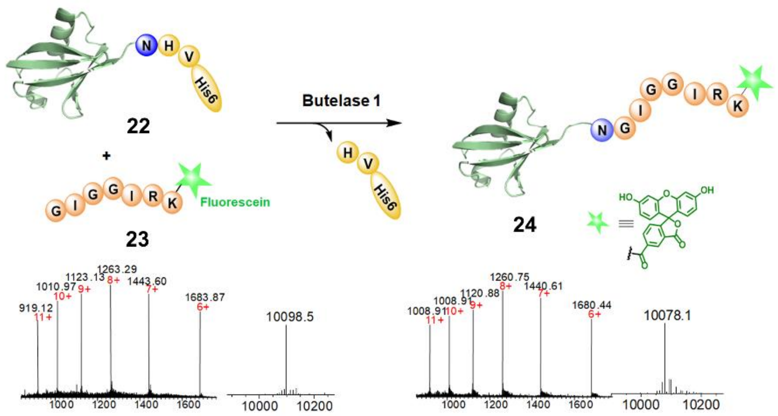


**Figure S8.** Ubiquitin **22** labeling via butelase-mediated ligation. Ubiquitin **22** containing the C-ter NHVHis6 tag was reacted with the fluorescein-peptide **23** using butelase-1. ESI-MS data of ubiquitin **22** calcd mass: 10097, found: 10098; The product **24** calcd mass: 10077 and found: 10078.

### f. IC$_{50}$ determination using MTT cell viability assay

The two types of cells, MCF-7 and A431, were both treated with peptide **11** or protein **12** at different concentrations: 0, 5,15, 25, 100, 200, 300, 400, and 1000 μM of **11** and 0, 0.25, 0.5, 1, 5, 20, and 30 μM of **12** for 84 h. Next, a MTT-based viability test was performed to determine the optical absorbance. Assays at each concentration point were run in triplicate. The IC$_{50}$ was calculated from the cell survival (%) *vs.* log (drug concentration) curves via non-linear regression method using Prism GraphPad.

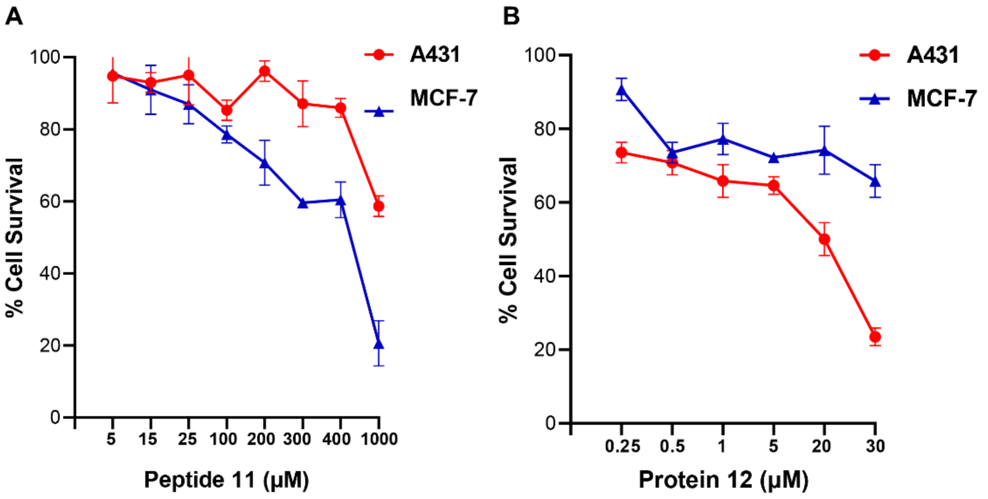


**Figure S9.** $IC_{50}$ calculation of MCF-7 and A431 cells. The cell survival curves of A431 and MCF-7 were both treated with A) peptide **11**; B) protein **12**.

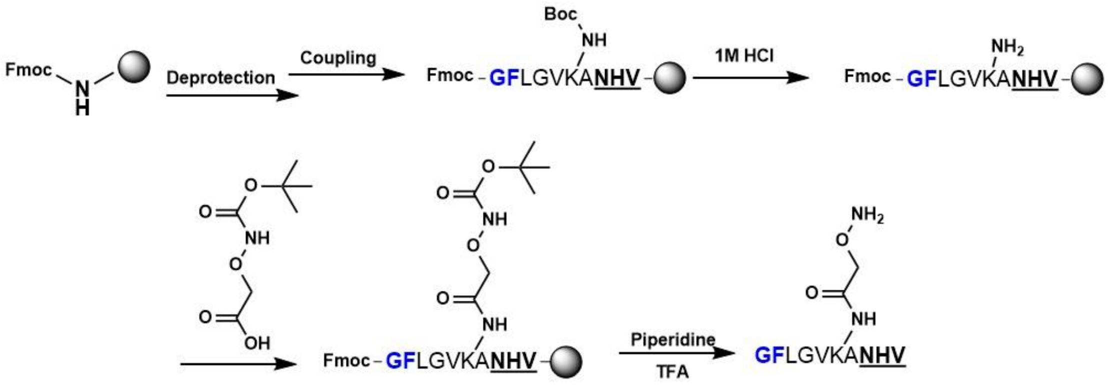


**Figure S10.** Synthesis of peptide **25**.

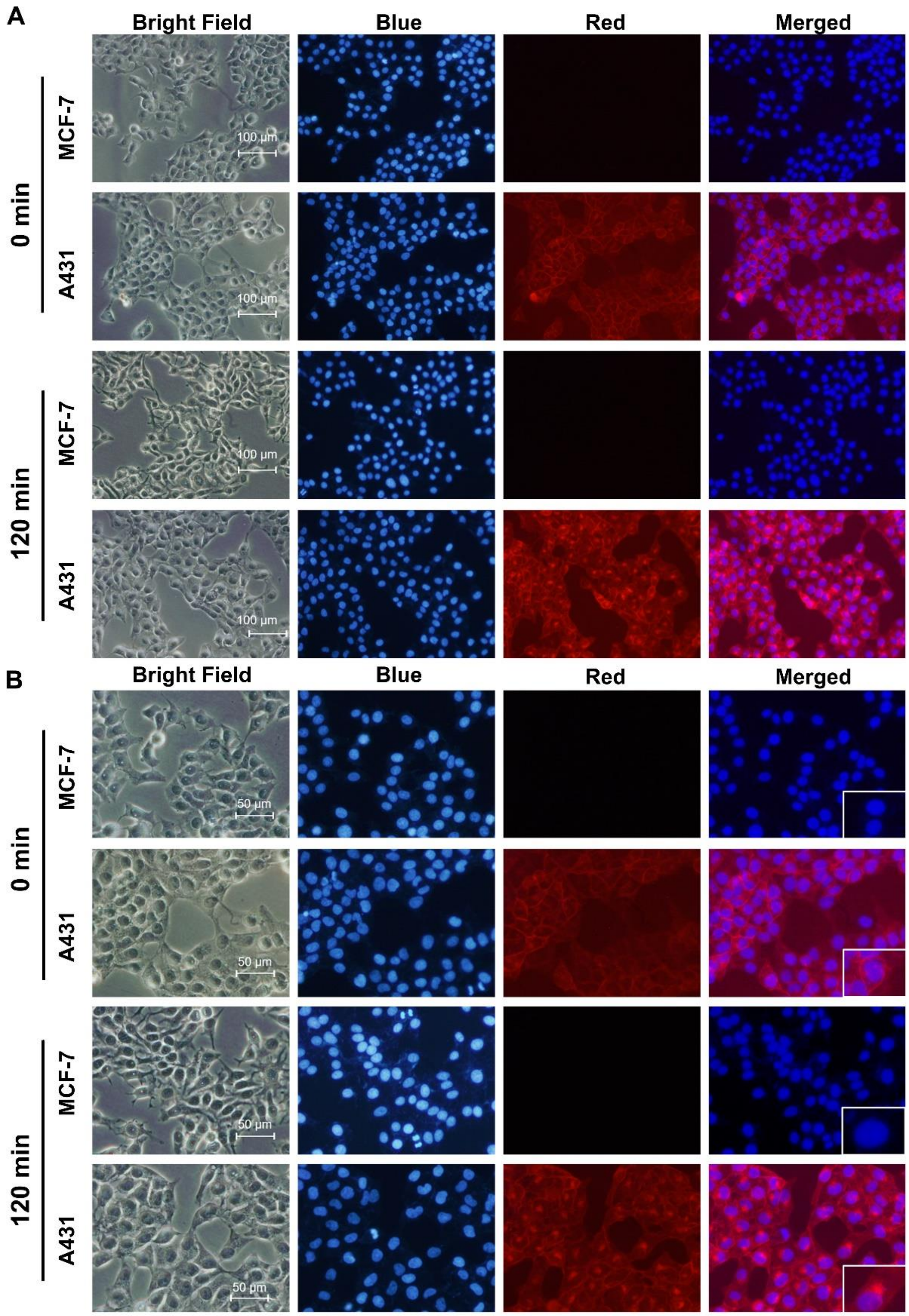


**Figure S11.** Cell imaging by the cyclic affibody-dox conjugate **30** after 120 min treatment. No clear cytotoxic effect was observed at this time. A) 20× magnification fluorescent microscopy analysis of MCF-7 cells before and after treatment with 2 μM of protein **30**. Scal bar, 100 μm; B) 32× magnification fluorescent microscopy analysis of MCF-7 cells before and after treatment with 2 μM of protein **30** for 120 min. Nucleus was stained with 700 nM of DAPI. Scale bar, 50 μm.

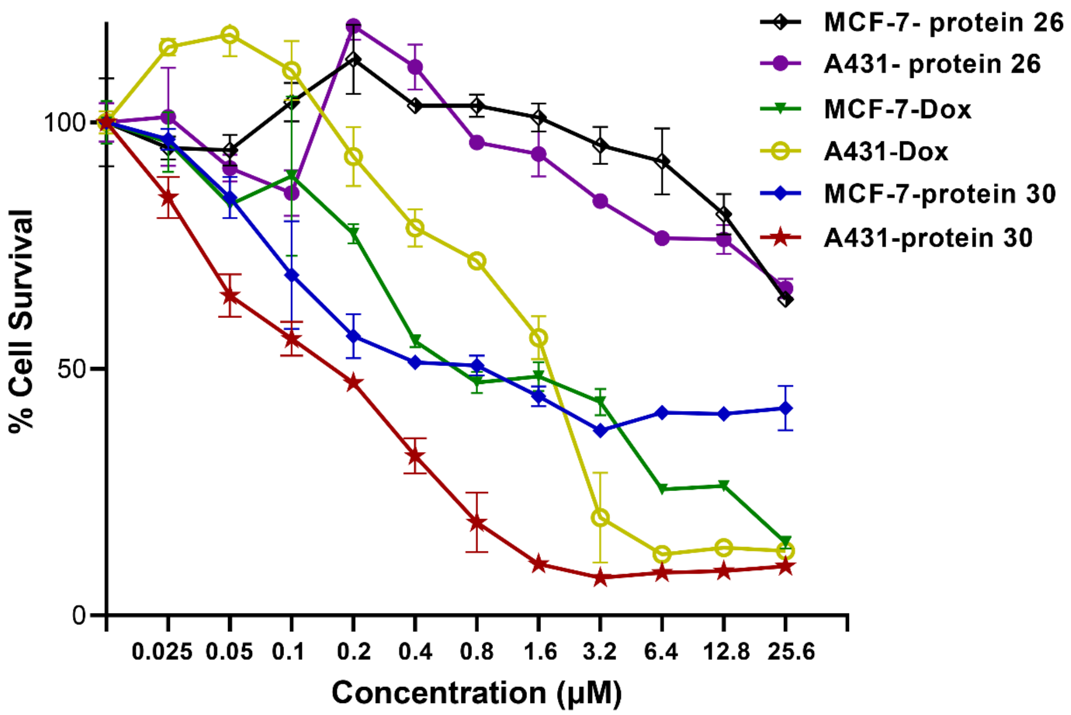


**Figure S12.** Cytotoxicity of various compounds on MCF-7 and A431 cells. Cells were treated with free doxorubicin, affibody **26**, and **30**, respectively, for 96 h. Then, a MTT-based viability test was performed to determine the optical absorbance to calculate the corresponding $IC_{50}$.

## 3. Mass spectra and HPLC profiles

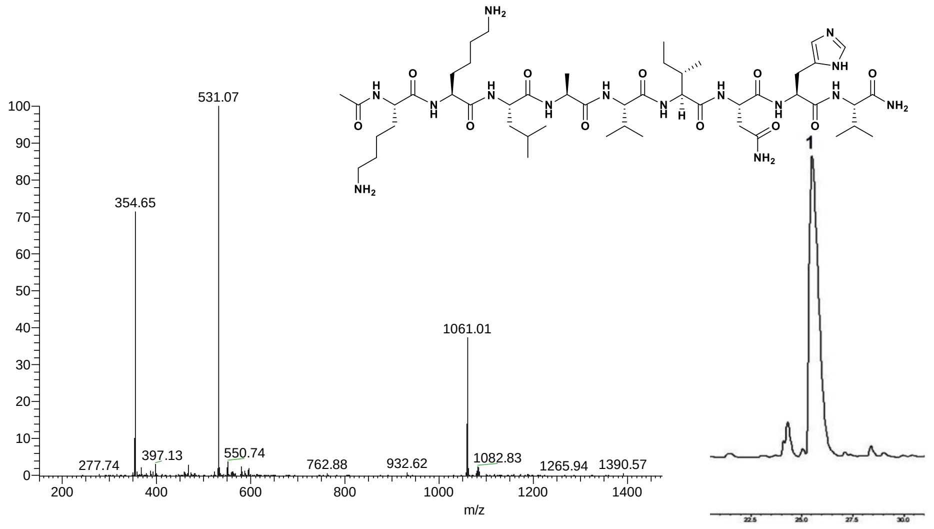


**Figure S13.** HPLC and ESI-MS of peptide **1**. ESI-MS of peptide **1:** 1061.01 (observed), 1062.27 (calculated).

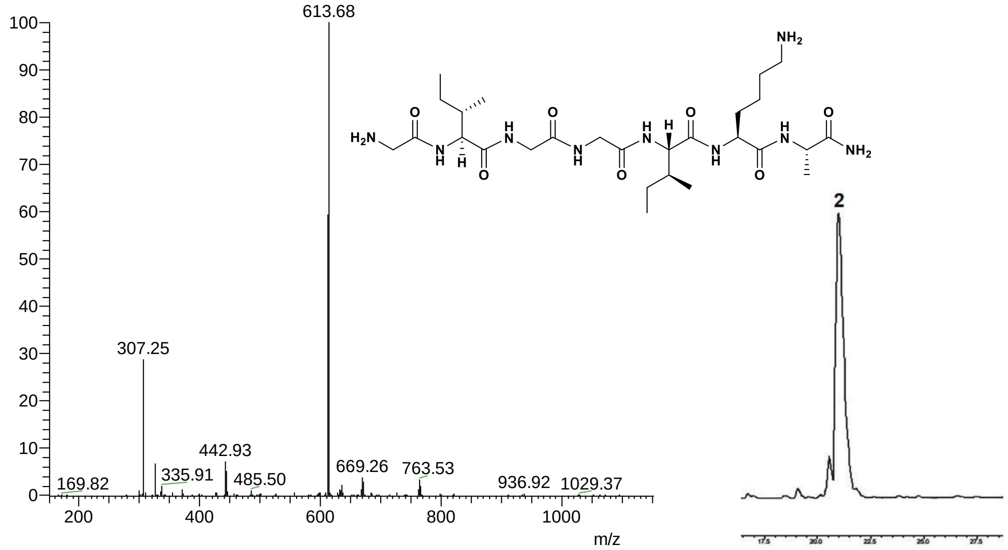


**Figure S14.** ESI-MS and HPLC of peptide **2**. ESI-MS of peptide **2:** ESI-MS: 613.68 (observed), 613.76 (calculated).

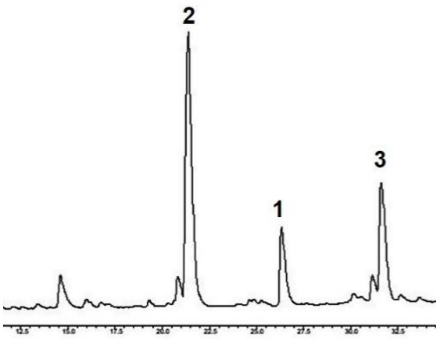


**Figure S15.** HPLC monitoring of the ligation between peptide **1** and **2** using butelase-1 to yield peptide **3** in kinetic studies.

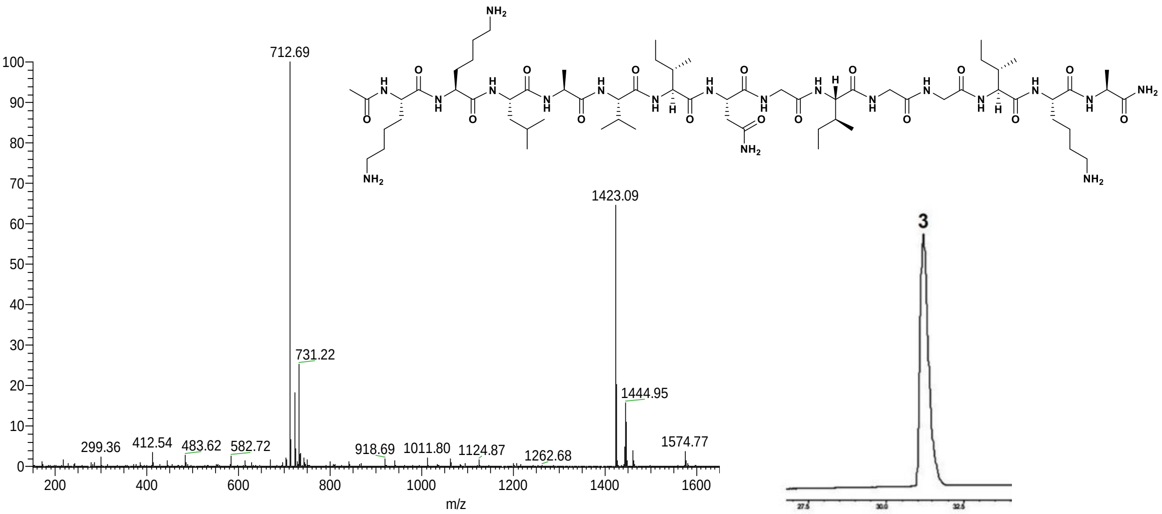


**Figure S16.** ESI-MS and HPLC of Peptide **3**. ESI-MS of peptide **3:** ESI-MS: 1423.09 (observed), 1422.78 (calculated).

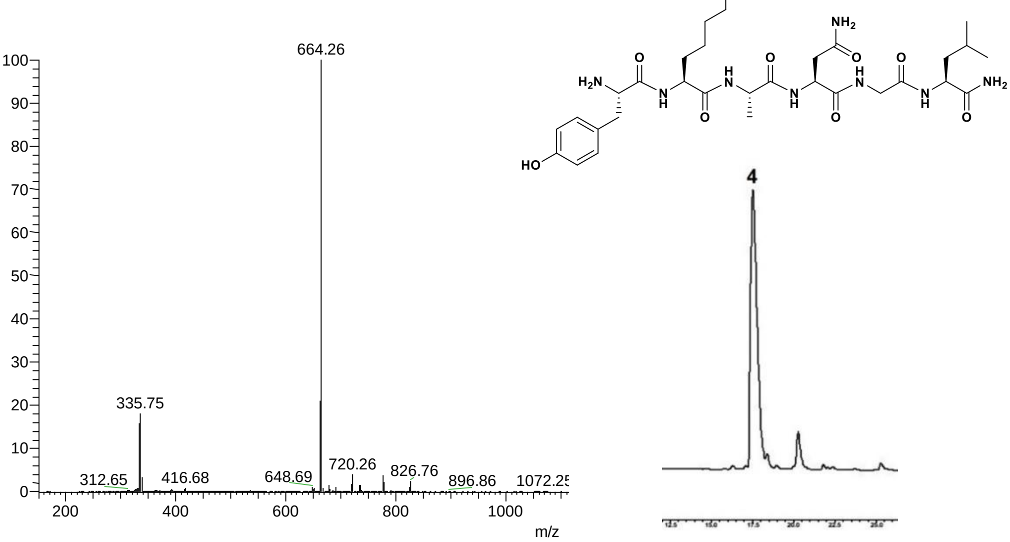

**Figure S17.** ESI-MS and HPLC of peptide **4**. ESI-MS of peptide **4:** ESI-MS: 664.26 (observed), 663.78 (calculated).

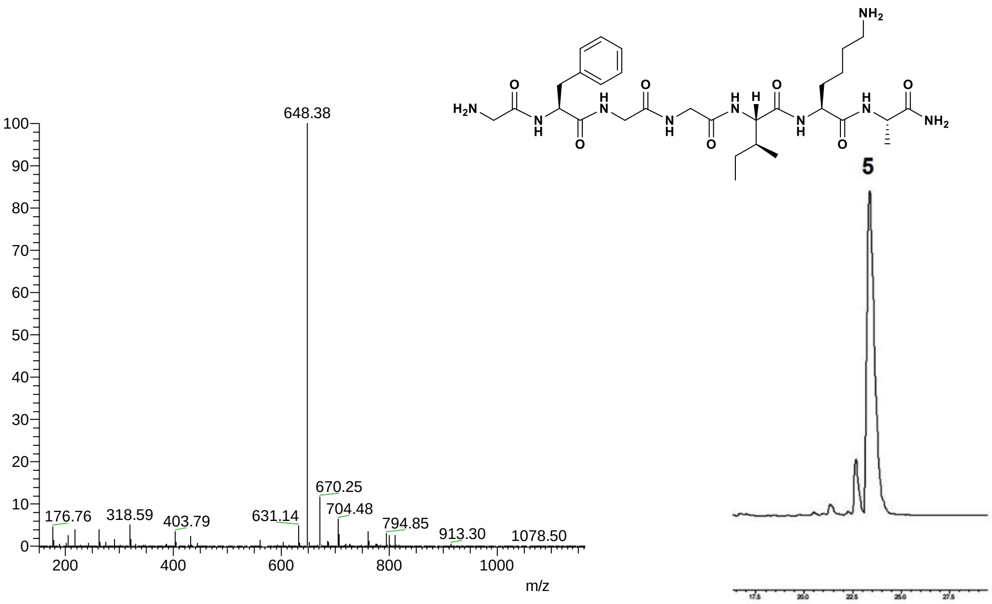


**Figure S18.** ESI-MS and HPLC of peptide **5**. ESI-MS of peptide **5:** ESI-MS: 648.38 (observed), 647.78 (calculated).

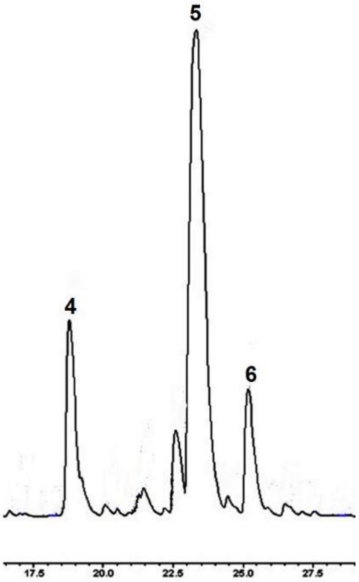


**Figure S19.** HPLC monitoring of the ligation between peptide **4** and **5** to yield peptide **6** using VyPAL2 in 5 min for kinetic studies.

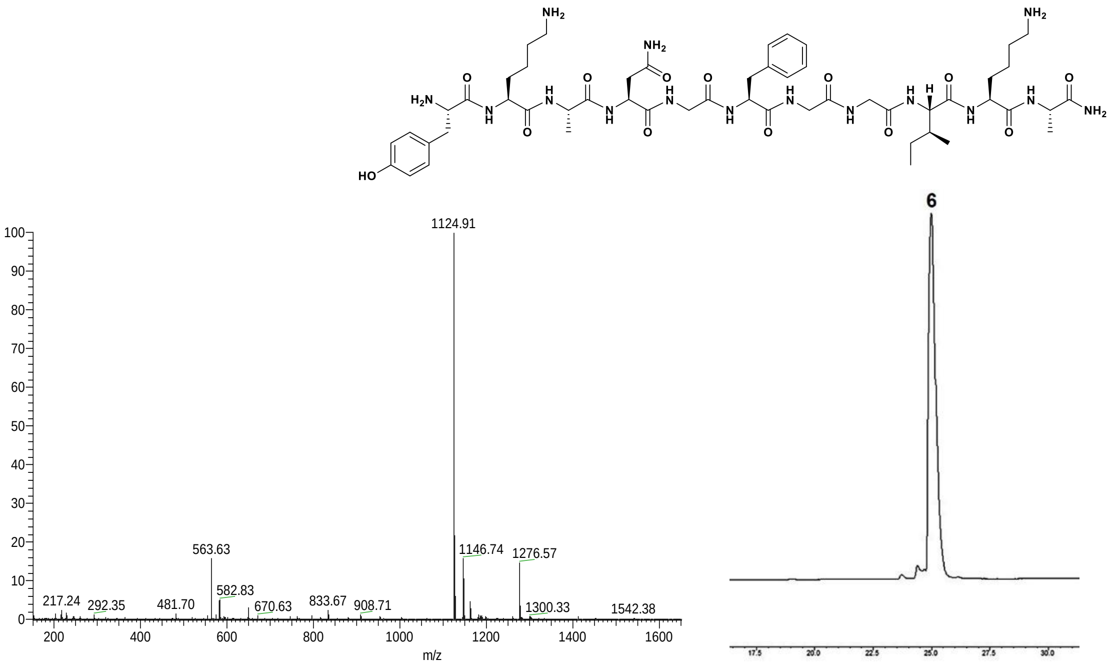


**Figure S20.** ESI-MS and HPLC of peptide **6**. ESI-MS of peptide **6:** ESI-MS: 1124.91 (observed), 1124.31 (calculated).

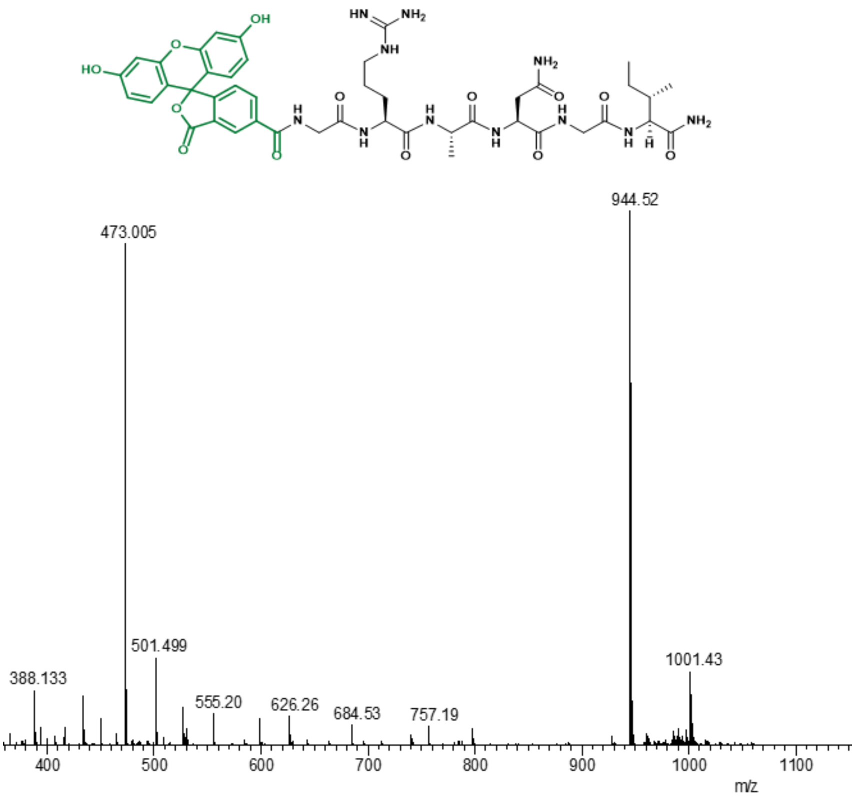


**Figure S21.** ESI-MS and HPLC of peptide **9**. ESI-MS of peptide **9:** ESI-MS: 944.52 (observed), 943.97 (calculated).

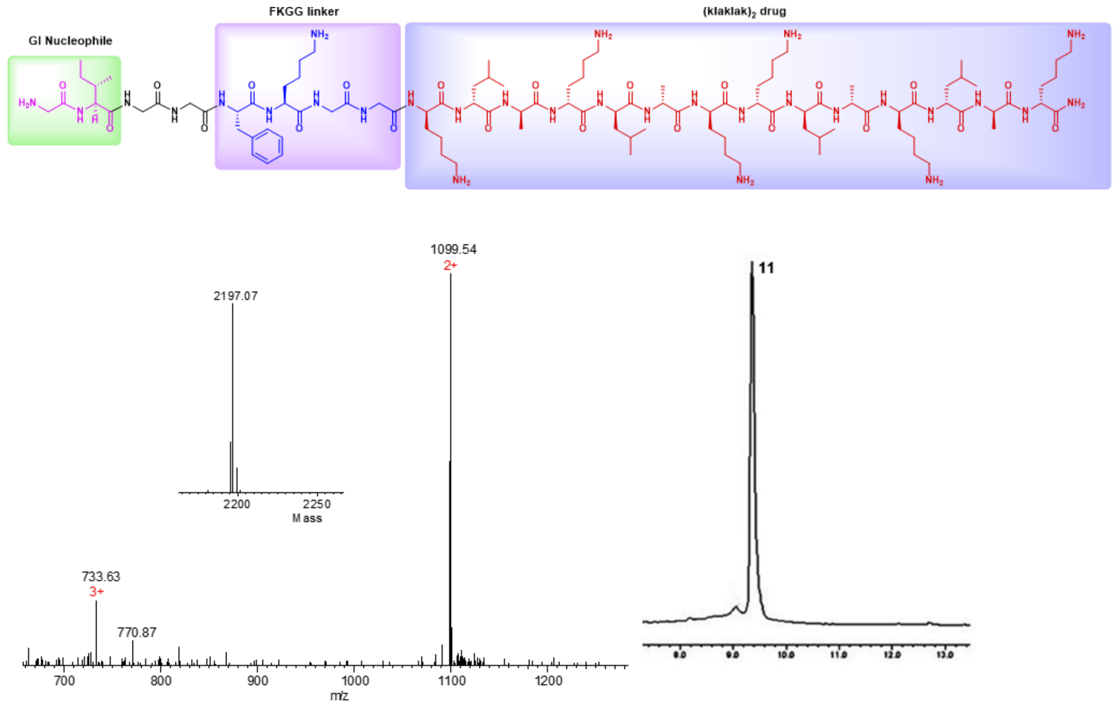


**Figure S22.** ESI-MS and HPLC of peptide **11**. ESI-MS of peptide **11:** ESI-MS: 2197.07 (observed), 2196.81 (calculated).

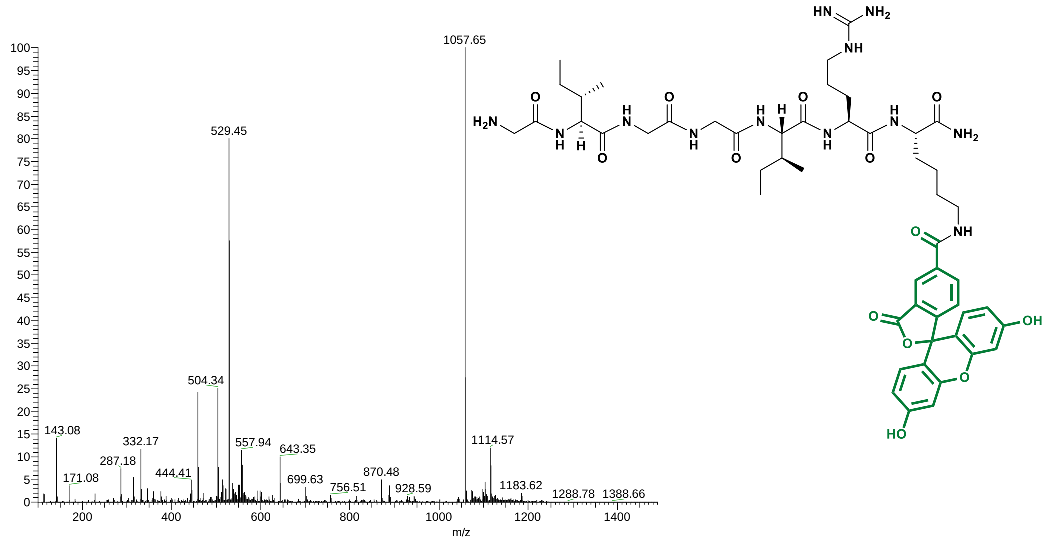


**Figure S23.** ESI-MS and HPLC of peptide **23**. ESI-MS of peptide **23:** ESI-MS: 1057.65 (observed), 1057.18 (calculated).

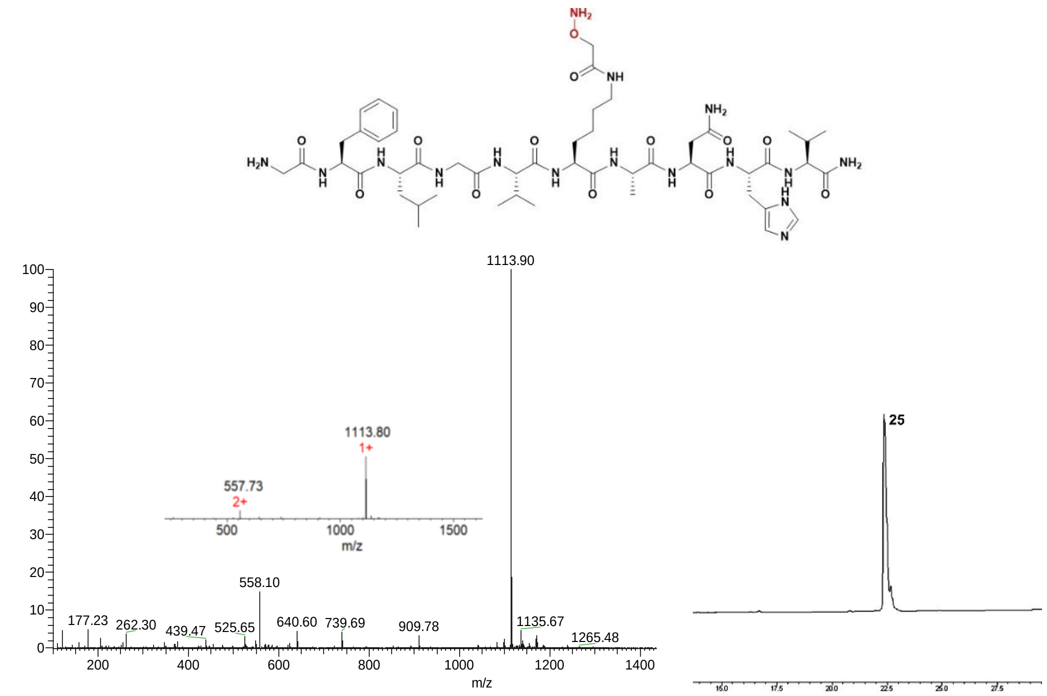


**Figure S24.** ESI-MS and HPLC of peptide **25**; ESI-MS of peptide **25:** ESI-MS: 1113.90 (observed), 1113.29 (calculated).
